# RNA Sequencing Reveals Upregulation of RUNX1-RUNX1T1 Gene Signatures in Clear Cell Renal Cell Carcinoma

**DOI:** 10.1155/2014/450621

**Published:** 2014-03-25

**Authors:** Zuquan Xiong, Hongjie Yu, Yan Ding, Chenchen Feng, Hanming Wei, Sha Tao, Dan Huang, Siqun Lilly Zheng, Jielin Sun, Jianfeng Xu, Zujun Fang

**Affiliations:** ^1^Department of Urology, Huashan Hospital, Fudan University, 12 Central Urumqi Road, Shanghai 200040, China; ^2^Fudan-VARI Center for Genetic Epidemiology, School of Life Sciences, Fudan University, Shanghai 200433, China; ^3^Laboratory of Bioinformatics, Center for Genetic Epidemiology and Prevention, Van Andel Research Institute, 333 Bostwick Avenue NE, Grand Rapids, MI 49503, USA; ^4^Center for Cancer Genomics, Wake Forest University School of Medicine, Medical Center Boulevard, Winston-Salem, NC 27157, USA; ^5^Laboratory of Computational, Laboratory of Genomics and Prevention, Center for Genetic Epidemiology and Prevention, Van Andel Research Institute, 333 Bostwick Avenue NE, Grand Rapids, MI 49503, USA

## Abstract

In the past few years, therapies targeted at the von Hippel-Lindau (VHL) and hypoxia-inducible factor (HIF) pathways, such as sunitinib and sorafenib, have been developed to treat clear cell renal cell carcinoma (ccRCC). However, the majority of patients will eventually show resistance to antiangiogenesis therapies. The purpose of our study was to identify novel pathways that could be potentially used as targets for new therapies. Whole transcriptome sequencing (RNA-Seq) was conducted on eight matched tumor and adjacent normal tissue samples. A novel RUNX1-RUNX1T1 pathway was identified which was upregulated in ccRCC through gene set enrichment analysis (GSEA). We also confirmed the findings based on previously published gene expression microarray data. Our data shows that upregulated of the RUNX1-RUNX1T1 gene set maybe an important factor contributing to the etiology of ccRCC.

## 1. Introduction 

Renal cell carcinoma (RCC) is one of the most common malignancies with the highest mortality rate among genitourinary cancers. Approximately 65,000 people were diagnosed and 14,000 deaths were attributed to cancers of the kidney and renal pelvis in 2010 in the United States [1]. While kidney cancer can be divided into several histological subtypes, the majority of the cases (about 75%) are clear cell renal cell carcinoma (ccRCC) [2]. Surgery offers the best opportunity to cure localized ccRCC. In the past few years, therapies targeted at VHL/HIF pathways, such as sunitinib and sorafenib, have been developed to treat ccRCC. However, most patients who either experience recurrence after surgery or have metastatic disease at the time of diagnosis will ultimately succumb to the disease. Thus, there remains a great need for novel therapies that depend on the identification of novel pathways in individuals with ccRCC.

Gene expression profiling, based on microarray hybridization, has been successfully used for the identification of genes that are differentially expressed among RCC subtypes and in the search for new therapeutic targets [3–6]. This method has also been correlated with chromosomal abnormalities and deregulated oncogenic pathways. However, the complimentary deoxyribonucleic acid cDNA microarray technique suffers from its inherent high background signals and depends on predesigned probes against known target transcripts, which makes it unable to detect novel transcript regions and it can only cover a portion of annotated transcriptome. The global detection of whole transcriptome is now possible with the recent development of next generation high-throughput RNA sequencing techniques (RNA-Seq). RNA-Seq has high technical and biological reproducibility. In addition, researchers have found RNA-Seq to be a powerful tool for the detection of differentially expressed genes, rare transcripts, novel isoforms, and mutations in tissues [7–10].

In this study, we performed whole transcriptome sequencing on eight pairs of ccRCC tumor and adjacent normal tissues in a Chinese population. Our goal was to identify novel gene pathways that have altered expression by comparing the expression patterns between the tumor and adjacent normal samples.

## 2. Materials and Methods

### 2.1. Patients and Samples

A total of 16 patients were treated with radical nephrectomy (RN) for RCC at Huashan Hospital of Fudan University. The 11 men and 5 women had a median age of 55 years (range of 44 to 75 years). Histological characterization for tumor type, such as ccRCC, was determined according to the Heildelberg classification, and staging was based on the American Joint Committee on Cancer (AJCC) TNM 2009 system. Twelve patients in the study group had pT1N0M0 tumors, two had T2N0M0 tumors, and the remaining two had T3N0M0 tumors. Clear cell renal cancer tumor and adjacent normal tissues were obtained from all 16 patients and a total of 16 pairs of tumor and adjacent normal tissues were available, from which 8 pairs of specimens were randomly selected for RNA sequencing to perform the gene profiling. Tumor tissues were selected from sites with high density of cancer without necrosis and normal tissues were sampled where no cancer contamination was found. All 16 pairs of samples were used to validate the genes differentially expressed between tumor and normal samples by quantitative real-time reverse transcription polymerase chain reaction. Specimens were frozen in liquid nitrogen immediately after operation and stored at −80°C. Detailed information of the study population was described in Table 1 and Supplementary Table 1 (see Supplementary Material available online at http://dx.doi.org/10.1155/2014/450621). The study was approved by the Institutional Review Board at Huashan Hospital of Fudan University, and all patients signed an informed consent form for inclusion of their samples.

### 2.2. cDNA Library Construction and Sequencing

Total RNA was isolated from frozen tumor and matched normal tissues using the reagent Trizol (Invitrogen). The sequencing library was constructed according to Illumina's TruSeq RNA Sample Preparation Protocol. Poly-A containing mRNA was purified from total RNA using magnetic beads with oligo-dT, followed by fragmentation. First-strand cDNA was synthesized using random hexamers and reverse transcriptase. Second-strand cDNA was synthesized with high quality deoxyribonucleotide triphosphates (dNTPs), ribonuclease H (RNase H), and DNA polymerase. Then the new double-strand cDNA was end-repaired and a single nucleotide “A” was added. Different in-house designed 6 bp adapters were ligated to the corresponding samples. DNA fragments with selected size and adapters were purified and amplified by PCR. After normalization, the DNA sample libraries were pooled into 4 libraries, and the pooled libraries were sequenced on an Illumina HiSeq 2000 sequencing machine.

### 2.3. Reads Mapping

Reads were processed and aligned to the University of California Santa Cruz (UCSC) H. sapiens reference genome and transcriptome (build hg19) using the RNA-Seq unified mapper (RUM) v1.0.9 [11]. RUM is an alignment program that maps reads in three phases. All reads were respectively aligned to the reference genome and transcriptome using Bowtie (v.12.7) [12]. The unmapped reads were then mapped to genome sequence with BLAT tool. Data collected from all the three mappings was then combined into a single mapping. This leverages the advantages of both genome and transcriptome mappings as well as combining the speed of Bowtie with the sensitivity and flexibility of Blat. The default parameters for RUM were used and more than 3 mismatches were allowed in the alignment. Finally, we used the Samtools software package (v0.1.17) to change the output of Bowtie to sorted bam files, which were used for further analysis [13].

### 2.4. Differential Expressed Gene (DEG) Analysis

The annotation for the UCSC known genes dataset was used for DEG data analysis for known genes. The exon union method was used to estimate the counts for each gene [14]. The genes differentially expressed between normal and tumor samples were then identified using negative binomial test as stated in the DESeq package [15]. Briefly, to compare differentially expressed genes between tumor and normal RCC samples, read counts of each of the identified genes were normalized to the total number of reads. The statistical significance (*P* value) was inferred based on the Bayesian method, a method specifically developed for the analysis of digital gene expression profiles and could account for the sampling variability of tags with low counts. A specific gene was deemed to be significantly differentially expressed if the *P* value given by this method was ≤0.05.

### 2.5. Pathway Analysis for RNA-Seq Data

The products of the negative log transformed *P* values, based on DEseq analysis plus the sign of the log2-fold change of each gene, were used as input to perform gene set enrichment analysis (GSEA) as implemented in the limma package. Specifically, the permutation test was used. The gene sets based on published papers were either generated in our lab or downloaded from the Molecular Signature Database (MsigDB, http://www.broadinstitute.org/gsea/msigdb/). These gene sets were curated from multiple sources including online pathway databases, biochemical literature, and mammalian microarray studies. Our main analysis was performed based on a class 2 database, which contains about 3,600 gene sets, which were curated from experimental data. The *P* values for each gene set were used to rank the functional representation of the significant genes in each gene set by their significance to the list of targets, thereby identifying biological processes likely to be affected.

### 2.6. Pathway Analysis for Microarray Data: PGSEA Analysis

Microarray databases (i.e., GSE17895 and GSE11024) were downloaded as described in the Section 3 and parameter gene set enrichment analysis (PGSEA) was used to generate enrichment scores for each pathway within each tumor sample using corresponding nondiseased kidney tissue as a reference [16]. A moderated *t*-statistic as implemented in the limma package was used to identify gene set enrichment scores that could discriminate between subtypes [5, 17].

### 2.7. Quantitative RT-PCR

Total RNA was isolated from tumor or adjacent normal tissues, followed by reverse transcription to cDNA using universal primers and a TaqMan Gene Expression Cells-to-CT Kit (Applied Biosystems). The qRT-PCR reaction was performed as previously described [18]. The primer sequences used for qRT-PCR were available upon request. *β*-Actin gene was used as internal quantitative control, and each assay was done in triplicate.

## 3. Results

### 3.1. RNA Sequencing

RNA sequencing was performed on eight matched pairs of ccRCC tumor and adjacent normal tissues. We generated an average of 14,306,899 (range: 3,186,698–27,215,607) single end reads with length of 100 bp, including 6 bp barcode sequence. The median total raw reads for the normal and tumor samples were approximately 11.8 million and 16.3 million separately, and the median alignment rates for the normal and tumor samples were 93.01% and 92.48% separately (Supplementary Table 2 and Supplementary Table 3).

We then quantified gene expression values in reads per kilobase of exon model per million mapped reads (RPKM) and observed that 56.30% of normal tissues and 55.48% of tumor tissues were less than 0.25 RPKM. In addition, approximately 1.11% and 1.12% of the genes separately for normal and tumor tissues were more than 5 RPKM (Supplementary Table 4). This suggests that there were only a very small number of genes expressed in relative high copies in those samples.

### 3.2. RNA-Seq Reveals Known DEG Changes in ccRCC

Using a cutoff of 0.05 for false discovery rate (FDR), we found that 3,514 (17.8%) out of 19,776 known genes were differentially expressed in tumor samples, including 2,054 (10.4%) upregulated genes and 1,460 (7.4%) downregulated genes. The top upregulated and downregulated genes are summarized in Table 2. Known HIF transcription targets, such as EGLN3 [19, 20], CA9 [21, 22], and VEGFA [23, 24], were in the top upregulated lists (Table 2). In addition, a set of known kidney differential markers such as KCNJ1 [25] and SLC22A8 were found in the top downregulated genes (Table 2). The top DEG list indicates that our whole transcriptome analysis recapitulates the known gene expression changes in ccRCC tumors.

### 3.3. RNA-Seq Reveals Novel RNX1/RNX1T1 Pathway/Signature Upregulated in ccRCC

A total of 206 pathway/signatures were upregulated in tumor samples and 23 pathway/signatures were downregulated in tumor samples compared with those in normal samples (Supplementary Table 5), using a *P* value cutoff of 0.001. The 35 upregulated and 2 downregulated pathways with most biological implications were selected and the relative deregulation of each gene sets for individual samples based on the enrichment *P* values were shown in Supplementary Figure 1.

Again, the known VHL/HIF pathway related gene sets were upregulated in tumor samples (Supplementary Figure 1). The gene sets related to kidney function were found downregulated in tumor samples, which indicates the loss of normal kidney function in these tumor samples. Both of these results showed the validity of our analytic approach.

**Figure 1:**
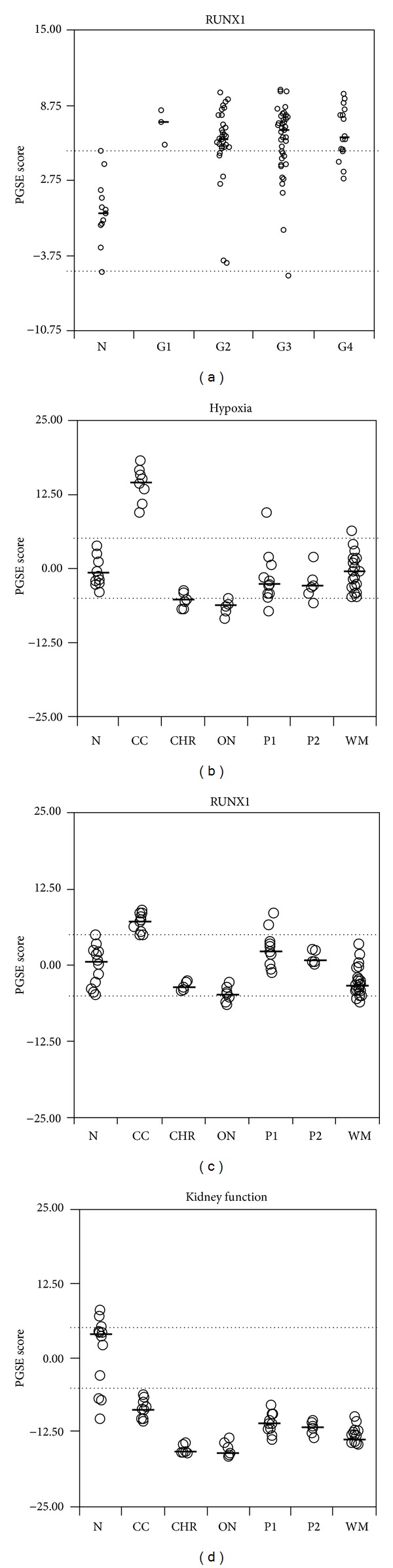
RUNX1-RUNX1T1 signature was upregulated in ccRCC. (a) PGSEA score from microarray data comprising 90 tumor and 13 normal tissues shows RUNX1-RUNX1T1 signature is up-regulated in ccRCC and shows no correlation to tumor grade. (b) Hypoxia related signature was specifically upregulated in ccRCC, and no significant upregulation was found in other kinds of RCC; (c) RUNX1-RUNXT1 related signature was specifically upregulated in ccRCC, and no significant upregulation was found in other kinds of RCC; (d) kidney function related signature was downregulated among all subtypes of RCC. N, normal samples; G1, tumor grade1; G2, tumor grade1; G3, tumor grade4; CC: clear cell; CHR: chromophobe; ON: oncocytoma; P: papillary; WM: Wilms' tumor.

The RUNX1-RUNX1T1 pathway was a novel gene set implicated in the pathway analysis. We then performed* in silico* confirmation study using a cohort with microarray data. The cohort included 90 ccRCC tumor tissue samples and 13 normal adjacent tissue samples [6]. Using PGSEA analysis, we found that RUNX1-RUNX1T1 was also upregulated in most of these 90 ccRCC cases and the upregulation of RUNX1-RUNX1T1 is not related to tumor grades (Figure 1(a)).

A total of 66 genes of the RUNX1-RUNX1T1 pathway were differentially expressed. We plotted the relative gene expression levels of 66 genes which were differentially expressed between tumor and normal tissues in the eight paired ccRCC samples based on RNAseq (Supplementary Figure 2(a)). The top 5 upregulated genes are NETO2, GBP2, VCAN, SLC1A3, and PLK2. The expression levels of these 66 genes from 90 ccRCC microarray data showed the same pattern of expression of these genes in tumor tissues (Supplementary Figure 2(b)). Three genes, including NETO2, GBP2, and VCAN, showed consistent high expression across these 90 tumor samples.

To determine whether the RUNX1-RUNX1T1 gene sets were specifically upregulated in the clear cell subtype, we performed another* in silico* study based on a second cohort of microarray data which included the most common subtypes of kidney cancers, including 27 Wilms' tumor, 10 ccRCC, 6 chromophobe, 7 oncocytoma, 17 papillary renal cell carcinoma, and 12 normal kidney samples [26]. The upregulation of VHL/HIF and RUNX1-RUNX1T1 gene sets was only observed in the clear cell subtype (Figures 1(b) and 1(c)). As a control measure, kidney function gene sets were lost in all of the subtypes of kidney cancers (Figure 1(d)). These results suggest that RUNX1-RUNX1T1 gene sets were specifically upregulated in ccRCC.

### 3.4. Validation of RUNX1-RUNX1T1 Genes in ccRCC by qRT-PCR

**Figure 2:**
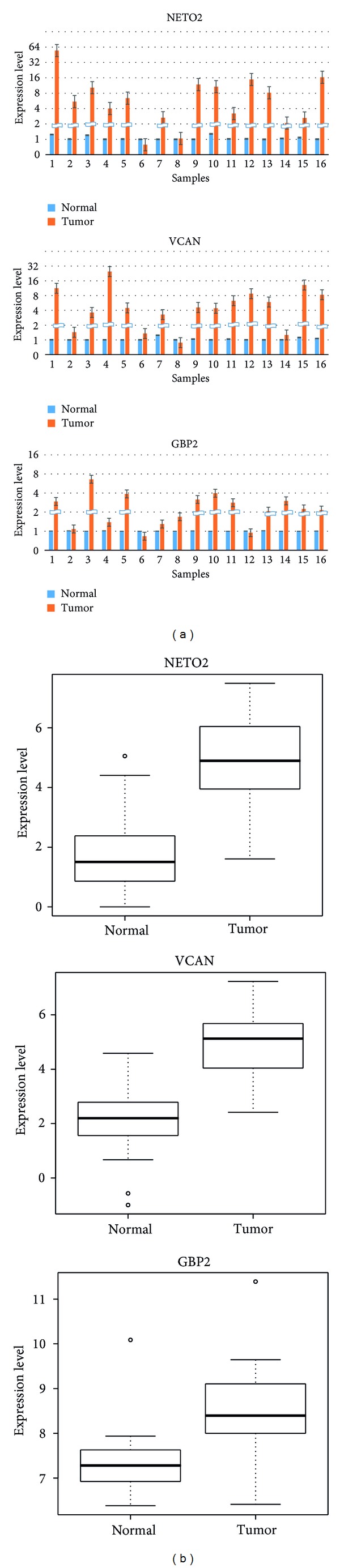
Validated upregulation of three RUNX1-RUNX1T1 genes in ccRCC by qRT-PCR. (a) Expression level of NETO2, VCAN, and GBP2 in 16 pairs of ccRCC tumor and normal tissues, including 8 pairs used for RNA-Seq and additional 8 pairs. *β*-Actin gene was used as reference gene. (b) Comparison of the expression level of NETO2, VCAN, and GPB2 between ccRCC tumor and adjacent normal tissues by qRT-PCR.

To confirm the upregulation of RUNX1-RUNXT1 genes in ccRCC, we examined the expression level of three upregulated genes of* NETO2*,* VCAN,* and* GBP2* in 16 pairs of ccRCC tumors and adjacent normal tissues using quantitative real-time PCR (qRT-PCR), including 8 pairs used for RNA sequencing and an additional 8 pairs of ccRCC tumor and normal tissues. The results of qRT-PCR were highly consistent with the RNA sequencing results (Figure 2). Overexpression of NETO2, VCAN, and GBP2 in tumor tissues compared to normal tissues were observed in 93.75% (15/16), 93.75 (15/16), and 87.5% (14/16), respectively.

## 4. Discussion

In the current study, we have applied next generation sequencing technology along with RNA-Seq data in eight matched tumor plus normal tissues to identify novel pathways for ccRCC. We have successfully identified a novel gene set for the fusion transcription factor, RUNX1-RUNX1T1, which is upregulated in ccRCC. We have also replicated the findings based on previously published gene expression microarray data.

Our study has been the first to establish a role of RUNX1-RUNX1T1 gene set in the carcinogenesis of ccRCC. A previous functional study showed that this fusion transcription factor disrupted the natural function of transcription factor RUNX1 and induced altered expression of genes such as AP-1 [27]. RUNX1-RUNX1T1, also called AML1/ETO, is considered a leukemia-specific chimeric fusion transcription factor. It is one of the most common chromosomal translocations in acute myeloid leukemia (AML) subtype 2 and is found in about 12% of AML patients [28]. However, its association with ccRCC has yet been reported and, through our work and the database online, this fusion has not been found in ccRCC.

Although the direct clinical association between ccRCC and AML remains unclear, there are still some similarities in the response to chemotherapy in both diseases. Since the 1980s, ccRCC has been established as a prototype of a chemotherapy-resistant tumor [29]. Fojo et al. (1987) reported that the expression of the multiple drug resistant- (MDR-)associated glycoprotein on the surface of RCC cells might contribute to this feature [30]. It would be interesting if future studies could reveal an inherent relation between these two diseases.

As aforementioned, we have revealed the upregulation of NETO2, GBP2, and VCAN in ccRCCs (Supp. Figure 2). NETO2 encodes a predicted transmembrane protein containing two extracellular CUB domains followed by a low-density lipoprotein class A (LDLa) domain. Expression of this gene may be a biomarker for proliferating infantile hemangiomas. Thus far, its expression in kidney tissue remains unidentified, which makes it more elusive in the functional prediction. Likewise, expression of GBP2 in RCC has not been reported either. GBP2 belongs to the family of GTP-binding proteins with limited known function. Recently, it has been reported that GBP2 may be mediated by p53 and become a disease marker in esophageal cancer [31]. VCAN, however, has been previously reported to be upregulated in RCC. Moreover, VCAN is associated with proliferation, survival, apoptosis, and migration in a variety of malignancies. Investigation of these rarely studied genes and their interrelations in RCC appears attractive.

Recently, the Cancer Genome Atlas (TGCA) has released a comprehensive molecular profile of ccRCC with astronomical matrix of data [32]. Using the online analytic software developed by Memorial Sloan Kettering Cancer Center (http://www.cbioportal.org/public-portal/), we have externally validated our top 3 hits in the TCGA database, in which the expressions of NETO2, GBP2, and VCAN studied by RNA-seq were consistently upregulated, respectively, in ccRCC patients. Moreover, NETO2 and GBP were associated with worsened prognosis in the TCGA cohort, further signifying our findings as potential disease markers or targets in ccRCC.

Our study has limitations. First, selected candidate genes should have been validated by quantitative PCR (Q-PCR) and, if better, by western blots. In lieu of Q-PCR, we have incorporated 2 sets of independent array data and have validated our results in expanded cases* in silico* (see Section 3). These validations further support the extrapolation of our findings. Nonetheless, future PCR validation and subsequent functional analysis are warranted. Second, due to our limited sample size, the interpretation of our results could be skewed. We are at present collecting more tissue samples for further investigation.

In summary, our novel findings of upregulation of genes within RUNX1-RUNX1T1 signature in ccRCC indicate that this gene set is critical for the tumorigenesis of ccRCC. Additional functional studies are required to delineate their functions in ccRCC.

## Supplementary Material

All the patients listed in the table were treated with radical nephrectomy (RN) for RCC at Huashan Hospital of Fudan University. The tissues experimented in RNA-seq test were from the first 8 cases. And we used the tissues for all 16 cases in qRT-PCR examination.

## Figures and Tables

**Table 1:** Summary of 8 ccRCC patients for RNA sequencing.

Patients ID	Age	Sex	TNM	Tumor grade	Sample type	Sample ID
1	47	Male	T1bN0M0	2	Normal	HS1N
					Tumor	HS1C

2	58	Female	T1aN0M0	3	Normal	HS2N
					Tumor	HS2C

3	64	Male	T1bN0M0	2	Normal	HS3N
					Tumor	HS3C

4	44	Female	T1bN0M0	3	Normal	HS4N
					Tumor	HS4C

5	62	Male	T1aN0M0	1	Normal	HS5N
					Tumor	HS5C

6	61	Female	T1bN0M0	1	Normal	HS6N
					Tumor	HS6C

7	61	Male	T1aN0M0	1	Normal	HS7N
					Tumor	HS7C

8	48	Male	T1bN0M0	2	Normal	HS8N
					Tumor	HS8C

**Table 2:** Summary of selected genes of interest differentially expressed in ccRCC samples.

Symbol	Entr. ID	log2-fold change	*P* value	Notes
SLC6A3	6531	8.41	1.59*E* − 139	
CA9	768	7.78	6.24*E* − 82	Hypoxia
FGG	2266	7.42	8.98*E* − 62	
NDUFA4L2	56901	6.95	7.34*E* − 122	
EGLN3	112399	5.89	7.19*E* − 97	Hypoxia
LOC100131551	100131551	4.38	1.18*E* − 42	
C3	718	5.31	1.81*E* − 89	
CYP2J2	1573	5.53	2.41*E* − 88	
ANGPTL4	51129	5.63	3.39*E* − 87	Hypoxia
FCGR3A	2214	4.72	2.88*E* − 57	
NETO2	81831	4.43	5.52*E* − 49	RUNX1
UBD	10537	4.33	1.21*E* − 59	
CP	1356	4.47	1.80*E* − 67	
PLIN2	123	4.12	3.01*E* − 53	Myc
PVT1	5820	3.79	6.24*E* − 56	Activator
ENO2	2026	3.19	2.19*E* − 21	Hypoxia
VEGF-A	7422	3.15	3.48*E* − 30	Hypoxia
SLC1A3	6507	3.04	8.64*E* − 24	RUNX1
PLK2	10769	2.82	2.57*E* − 22	RUNX1
GBP2	2634	2.62	1.37*E* − 24	RUNX1
VCAN	1462	2.44	4.65*E* − 24	RUNX1
COL1A1	1277	2.31	1.03*E* − 22	SWI/SNF
SEL1L3	23231	1.84	5.38*E* − 13	SWI/SNF
BCAT1	586	1.83	7.89*E* − 08	SWI/SNF
SIM2	6493	−5.70	2.02*E* − 53	TF
SLC13A3	64849	−5.97	4.53*E* − 52	
SERPINA5	5104	−5.81	2.08*E* − 42	
SFRP1	6422	−5.91	5.30*E* − 48	Wnt
KCNJ1	3758	−7.26	4.84*E* − 70	Kidney
SCNN1G	6340	−8.95	7.28*E* − 64	
TFAP2B	7021	−7.64	5.21*E* − 44	TF
KNG1	3827	−8.97	4.07*E* − 91	
SLC12A1	6557	−8.16	1.85*E* − 90	Kidney
HSFY2	159119	−7.41	4.54*E* − 42	TF
SLC22A8	9376	−8.10	1.94*E* − 64	Kidney
GP2	2813	−11.51	1.74*E* − 57	
AQP2	359	−11.64	9.68*E* − 118	Kidney
UMOD	7369	−12.12	3.25*E* − 127	Kidney
