# Supplementary material for: RNA Sequencing Reveals Upregulation of RUNX1-RUNX1T1 Gene Signatures in Clear Cell Renal Cell Carcinoma
Source: Biomed Res Int. 2014 Mar 25;2014:450621. doi: 10.1155/2014/450621 (PMC3982423; doi:10.1155/2014/450621)

Supplementary Table1. Summary of 17 ccRCC patients used for RNA sequencing and qRT-PCR

Patients ID	Age	Sex	TNM	Tumor Grade	Sample Type	Sample ID	RNA-seq	qRT-PCR
HS09-9020	47	Male	T1bN0M0	2	Normal	HS1N	Yes	Yes
					Tumor	HS1C		
HS09-9470	58	Female	T1aN0M0	3	Normal	HS2N	Yes	Yes
					Tumor	HS2C		
HS09-12586	64	Male	T1bN0M0	2	Normal	HS3N	Yes	Yes
					Tumor	HS3C		
HS09-14456	44	Female	T1bN0M0	3	Normal	HS4N	Yes	Yes
					Tumor	HS4C		
HS09-21660	62	Male	T1aN0M0	1	Normal	HS5N	Yes	Yes
					Tumor	HS5C		
HS10-1110	61	Female	T1bN0M0	1	Normal	HS6N	Yes	Yes
					Tumor	HS6C		
HS10-3063	61	Male	T1aN0M0	1	Normal	HS7N	Yes	Yes
					Tumor	HS7C		
HS10-2142	48	Male	T1bN0M0	2	Normal	HS8N	Yes	Yes
					Tumor	HS8C		
HS08-02026	51	Female	T1aN0M0	3	Normal	HS9N	No	Yes
					Tumor	HS9C		
HS08-20425	44	Male	T1bN0M0	3	Normal	HS10N	No	Yes
					Tumor	HS10C		
HS07-24040	55	Female	T3bN0M0	4	Normal	HS11N	No	Yes
					Tumor	HS11C		
HS07-22442	49	Male	T3bN0M0	3	Normal	HS12N	No	Yes
					Tumor	HS12C		
HS10-25343	62	Male	T1bN0M0	3	Normal	HS13N	No	Yes
					Tumor	HS13C		
HS11-02644	75	Male	T1aN0M0	2	Normal	HS14N	No	Yes
					Tumor	HS14C		
HS08-00671	44	Male	T2aN0M0	4	Normal	HS15N	No	Yes
					Tumor	HS15C		
HS08-16961	56	Male	T2aN0M0	2	Normal	HS16N	No	Yes
					Tumor	HS16C		

Supplementary Table 2. Summary of sequencing data for 8 paired ccRCC samples

	Sample Type	Total reads	Unique mappers	None Unique Mappers	Total Mappers
Median	Normal	11840674	66.31%	26.70%	93.01%
	Tumor	16265923	66.16%	25.21%	92.48%
Min	Normal	3186698	60.39%	23.05%	91.01%
	Tumor	6745117	55.49%	18.96%	90.81%
Max	Normal	27215607	69.13%	31.69%	93.87%
	Tumor	20788570	73.94%	36.23%	93.33%

Supplementary Table 3. Summary of sequencing data for each 8 paired ccRCC samples

Patients ID	Sample ID	Total reads	Unique mappers	None Unique Mappers	Total Mappers	Total Coverage
HS09-9020	HS1N	1E+07	6903999 66.31%	2780134 26.70%	9684133 93.00%	30.34
	HS1T	1.9E+07	1.3E+07 70.06%	4457233 23.20%	17879280 93.30%	56.02
HS09-9470	HS2N	3186698	1924600 60.39%	975459 30.60%	2900059 91%	9.08
	HS2T	6745117	4607839 68.31%	1592387 23.60%	6200226 91.90%	19.43
HS09-12586	HS3N	8646510	5907843 68.32%	2201839 25.40%	8109682 93.70%	25.41
	HS3T	9944835	6579550 66.16%	2617574 26.30%	9197124 92.40%	28.82
HS09-14456	HS4N	1.7E+07	1.1E+07 65.44%	4718113 27.50%	1.6E+07 92.90%	49.92
	HS4T	1.3E+07	7847820 60.39%	4017213 30.90%	1.2E+07 91.30%	37.18
HS09-21660	HS5N	8632224	5765287 66.78%	2290340 26.50%	8055627 93.30%	25.24
	HS5T	8156202	6030966 73.94%	1546596 18.90%	7577562 92.90%	23.74
HS10-1110	HS6N	2.1E+07	1.4E+07 65.99%	5631365 27.00%	1.9E+07 93.00%	60.71
	HS6T	1.6E+07	9026590 55.49%	5892618 36.20%	1.5E+07 91.70%	46.75
HS10-3063	HS7N	1.2E+07	7224634 61.01%	3752065 31.60%	1.1E+07 92.70%	34.39
	HS7T	1.9E+07	1.2E+07 65.59%	4769054 25.20%	1.7E+07 90.80%	53.82
HS10-2142	HS8N	2.7E+07	1.8E+07 67.75%	7106803 26.10%	2.6E+07 93.80%	80.04
	HS8T	2.1E+07	1.3E+07 63.78%	6054283 29.10%	1.9E+07 92.90%	60.52

Supplementary Table 4. Summary of reads counts data distribution for single gene for 8 paired ccRCC samples

Patients ID	Sample ID	Reads per Kilobase per Million Reads (RPKM)						
		>50	>5	>2	>1	>0.5	>0.25	0-0.25
Median	Normal	0.05%	1.11%	3.00%	6.28%	12.15%	21.06%	56.30%
	Tumor	0.08%	1.12%	3.13%	6.42%	12.45%	20.76%	55.48%
Min	Normal	0.02%	0.84%	2.46%	5.39%	11.29%	20.45%	52.26%
	Tumor	0.05%	1.04%	2.90%	5.97%	11.77%	20.24%	51.42%
Max	Normal	0.09%	1.64%	4.19%	7.40%	12.83%	22.30%	59.55%
	Tumor	0.14%	1.66%	3.77%	7.64%	13.68%	22.02%	57.83%
HS09-9020	HS1N	0.05%	1.09%	2.93%	6.28%	12.24%	21.11%	56.30%
	HS1C	0.05%	1.04%	2.90%	6.04%	12.44%	21.59%	55.94%
HS09-9470	HS2N	0.09%	1.64%	4.19%	7.40%	12.79%	21.62%	52.26%
	HS2C	0.12%	1.66%	3.77%	7.64%	13.44%	21.58%	51.80%
HS09-12586	HS3N	0.05%	1.11%	3.04%	6.34%	12.05%	21.06%	56.35%
	HS3C	0.08%	1.04%	2.91%	6.42%	12.56%	21.52%	55.48%
HS09-14456	HS4N	0.05%	1.22%	3.15%	6.28%	12.15%	20.88%	56.26%
	HS4C	0.14%	1.54%	3.63%	6.88%	12.58%	20.42%	54.81%
HS09-21660	HS5N	0.07%	1.16%	3.00%	6.41%	12.57%	22.04%	54.74%
	HS5C	0.11%	1.48%	3.71%	7.57%	13.68%	22.02%	51.42%
HS10-1110	HS6N	0.05%	1.14%	3.14%	6.33%	12.15%	20.83%	56.36%
	HS6C	0.06%	1.04%	2.99%	5.97%	11.77%	20.34%	57.83%
HS10-3063	HS7N	0.03%	0.84%	2.46%	5.39%	11.29%	20.45%	59.55%
	HS7C	0.07%	1.12%	3.13%	6.31%	12.32%	20.76%	56.28%
HS10-2142	HS8N	0.05%	1.10%	2.93%	5.98%	11.89%	20.77%	57.28%
	HS8C	0.10%	1.43%	3.47%	6.71%	12.45%	20.44%	55.40%

Sup table 5. Signatures significantly associated with ccRCC tumor and normal samples.

Systematic Name[a]	Standard Name	# genes in term	# of difference expressed gene[b]	Contribution genes[c]	Brief description[d]	Direction[e]	Padj[f]
M7363	ELVIDGE_HYPOXIA_UP	369	58	CADM1,HLA-DRB1,MXI1,ISG20,INSIG2,SLC2A1,TGFBI,ZNF395,LOX,SAMD4A,S100A2,EGFR,STC2,OLFML2A,DDIT4,SPAG4,VEGFA,STC1,HOXA7,CYP1B1,ALDOC,AKAP12,SFXN3,SOX9,DAAM1,OBSL1,AHNAK2,SCARB1,NDRG1,ERO1L,ANGPTL4,KLF6,EMR2,GJA1,BNIP3,SPRY1,HEY1,P4HA1,CXCR4,LOXL1,NOL3,PFKP,	Genes up-regulated in MCF7 cells (breast cancer) under hypoxia conditions.	up	0
M9197	ELVIDGE_HYPOXIA_BY_DMOG_UP	279	49	CADM1,HLA-DRB1,MXI1,ISG20,INSIG2,TGFBI,SLC2A1,ZNF395,LOX,SAMD4A,S100A2,EGFR,STC2,OLFML2A,DDIT4,SPAG4,VEGFA,STC1,ALDOC,AKAP12,SFXN3,SOX9,DAAM1,AHNAK2,OBSL1,NDRG1,ERO1L,ANGPTL4,KLF6,GJA1,BNIP3,SPRY1,HEY1,CXCR4,P4HA1,PFKP,CYB5A,ADM,CA9,PPFIA4,EGLN3,HK2,ENO2,	Genes up-regulated in MCF7 cells (breast cancer) treated with hypoxia mimetic DMOG [PubChem=3080614] .	up	0
M259	MANALO_HYPOXIA_UP	427	60	PGF,PTGS1,EDN1,MXI1,APOD,ZNF395,STC2,FER1L4,SPAG4,VEGFA,STC1,IDH2,LAMB1,SMAD6,NOTCH4,BNIP3,P4HA1,CHST15,TNFRSF10B,ADM,ARL4C,SPSB1,AXL,RASSF2,SLC2A3,TGFBI,LOX,FNDC3B,HLA-B,HLA-E,DDIT4,TNS1,COL1A2,TNFAIP3,FAM171A1,ABCA1,NDRG1,ERO1L,ANGPTL4,CUBN,CDKN1C,ALDH2	Genes up-regulated in response to both hypoxia and overexpression of an active form of HIF1A [GeneID=3091] .	up	0
M11033	MENSE_HYPOXIA_UP	203	33	OSMR,FAM110C,MXI1,INSIG2,SLC2A3,LOX,STC2,SPAG4,VEGFA,PFKFB4,ALDOC,PPP1R3C,NDRG1,ERO1L,ANGPTL4,SCD,NAMPT,BNIP3,SLC2A3,P4HA1,BHLHE41,LOC154761,NOL3,ADM,PPFIA4,HK2,PLIN2,ANKRD37,ENO2,PDK1,RNASE4,SOD2,PGK1	Hypoxia response genes up-regulated in both astrocytes and HeLa cell line.	up	0
M14072	WINTER_HYPOXIA_METAGENE	438	80	LDHB,LDHA,PTGS2,EFNA1,PGF,EDN1,PTGS1,PGAM1,SYT7,MXI1,STC2,KRT19,F3,ALDOA,PFKFB4,ALDOC,TPI1,LGALS1,KDR,IFI27,ETS1,PLAU,PPARA,BNIP3,P4HA1,TGFA,ANGPT2,ICAM1,MMP13,ADM,HLA-DQB1,HK2,MIF,THBS2,IGF2,LRP1,BIK,CP,PGK1,RPL36A,ENPEP,TGFB1,SLC16A1,SLC2A3,TGFBI,SLC2A1	Genes regulated by hypoxia	up	0
M3996	JIANG_HYPOXIA_NORMAL	444	51	ZNF395,STC2,UGCG,SIRPA,GRB10,NEK6,ALDOC,AKAP12,IFI30,ASB15,PEA15,PPP1R3C,OBSL1,KLF6,SCD,BNIP3,CANX,P4HA1,KCNMA1,PLIN2,SOD2,LRP1,NUPR1,SLC2A1,LOX,CA12,HIF1A,PI4K2A,MCL1,MDK,NDRG1,NDRG2,GAL3ST1,ANGPTL4,P4HB,CORO1A,FAM84A,HMOX1,ARHGAP8,TSKU,KHDRBS3,NOL3,SNX1	Genes up-regulated in RPTEC cells (normal kidney) by hypoxia.	up	0
M19622	LEONARD_HYPOXIA	70	16	ALDOA,PFKFB4,EFNA1,BNIP3,SLC7A5,SAP30,PLIN2,P4HA1,SLC2A3,SLC2A1,TGFA,MUC1,STC2,PFKP,DUSP1,F3	Genes up-regulated in HK-2 cells kidney tubular epithelium) under hypoxia and down-regulated on re-oxygenation.	up	0
M10508	HARRIS_HYPOXIA	160	40	LDHA,PTGS2,PGF,EDN1,BNIP3,L1CAM,ENPEP,TGFB1,SLC2A3,P4HA1,HMOX1,SLC2A1,TGFA,ANGPT2,CA12,PFKP,MMP13,HIF1A,ADM,CA9,F3,PKM2,STC1,ALDOA,VIM,HK2,MIF,TGM2,GAPDH,EPO,ENO1,FLT1,AK3,IGF2,CDKN1A,BIK,CP,PGK1,IGFBP2,IGFBP3	Genes known to be induced by hypoxia	up	0
M2513	ELVIDGE_HIF1A_TARGETS_DN	195	38	HLA-DRB1,BNIP3,GJA1,MXI1,ISG20,MAGED4,MAGED4B,INSIG2,P4HA1,CXCR4,SLC2A1,ZNF395,LOX,LOXL1,EGFR,NOL3,OLFML2A,ADM,CA9,SPAG4,STC1,PPFIA4,ALDOC,HK2,SFXN3,EGLN3,PLIN2,ENO2,AHNAK2,OBSL1,NDRG1,ERO1L,SCNN1B,ANGPTL4,PDK1,RNASE4,PGK1,IGFBP3	Genes down-regulated in MCF7 cells (breast cancer) after knockdown of HIF1A [GeneID=3091] by RNAi.	up	0
M6189	ELVIDGE_HIF1A_AND_HIF2A_TARGETS_DN	221	44	HLA-DRB1,MXI1,INSIG2,SLC2A1,ZNF395,LOX,SAMD4A,EGFR,SPAG4,VEGFA,STC1,ALDOC,AKAP12,SFXN3,SOX9,OBSL1,AHNAK2,NDRG1,ERO1L,ANGPTL4,GJA1,BNIP3,MAGED4,MAGED4B,HEY1,CXCR4,P4HA1,LOXL1,NOL3,CYB5A,ADM,CA9,PPFIA4,EGLN3,HK2,GPRC5A,PLIN2,ENO2,SCNN1B,PDK1,RNASE4,DPYSL2,P	Genes down-regulated in MCF7 cells (breast cancer) after knockdown of both HIF1A and HIF2A [GeneID=3091;2034] by RNAi.	up	0

M14973	SENESE_HDAC1_TARGETS_UP	953	63	OSMR,TFPI,ESM1,RAC2,ATP11A,RASSF8,ERRFI1,PRSS35,MYC,CCND2,G0S2,DEGS1,C3,GPR65,CCL20,BUB1,PHLDA1,PSAT1,TCFL5,NFE2L2,TNFAIP3,TNFRSF12A,SLC43A3,IFI16,COBLL1,IL6ST,VEGFA,LAMC2,SAA2,IGF2BP2,SLAMF7,ETS1,PLAU,CPEB4,TNFRSF11B,AGPAT9,TNFRSF10B,SLC39A14,PLIN2,BIRC3	Genes up-regulated in U2OS cells (osteosarcoma) upon knockdown of HDAC1 [GeneID=3065] by RNAi.	up	0
M8776	HELLER_SILENCED_BY_METHYLATION_UP	500	93	L1CAM,ISG20,PRAME,NCF2,OLFML2B,IFI44,HLA-DQA2,HLA-DQA1,SSTR2,CX3CR1,TFPI,ABAT,HLA-DPA1,LAMC2,HLA-DRA,GCNT3,CYP1B1,HLA-DRB1,ENPP2,XIST,AKAP12,HSPA1A,IDH2,HLA-DRB6,HLA-DPB1,HLA-DOB,CHI3L1,SLAMF7,MCAM,CEL,IFI27,RGS1,CLEC7A,PLA2G4C,MSR1,TOP2A,ICAM1,HLA-DQB1,P	Genes up-regulated in at least one of three multiple myeloma (MM) cell lines treated with the DNA hypomethylating agent decitabine (5-aza-2'-deoxycytidine) [PubChem=451668] .	up	0
M7623	HELLER_HDAC_TARGETS_SILENCED_BY_METHYLATION_DN	490	60	ISG20,RPL10,PITX1,CCR2,ST14,BTN3A2,HSPA1A,OAS1,OAS2,HSPA1B,SERPINH1,TCF4,BLNK,SCD,S100A11,CLEC7A,GJA1,MYC,CEACAM1,FMNL1,MYCN,CCND1,CCND2,FKBP11,MYOM2,CLEC2B,BCL2,PDK1,SHMT2,LRRC41,CD180,QPCT,PSAT1,RBM3,SLC7A5,CDKN2A,CDKN2C,DDIT4,SLC38A1,FGFR3,FXYD5,LAPTM5	Genes down-regulated in multiple myeloma (MM) cell lines treated with both decitabine [PubChem=451668] TSA [PubChem=5562] .	up	0
M12272	MISSIAGLIA_REGULATED_BY_METHYLATION_UP	200	33	ATP1B1,PTGS2,ISG20,HIST1H2BK,IL1B,BST2,MFI2,HLA-E,TACSTD2,COL1A1,TNFAIP3,TNFAIP2,OAS1,OAS2,TIMP1,PLAT,UBE2L6,ITGA2,LGALS9,CDKN1A,QPRT,UBD,PRSS8,CCL20,LY6E,TAP1,IGF2,BIRC3,STAT1,SOD2,DUSP1,SLC6A8,IGFBP3	Genes up-regulated in PaCa44 and CFPAC1 cells (pancreatic cancer) after treatment with decitabine [PubChem=451668]	up	3.00E-04
M18085	LIANG_SILENCED_BY_METHYLATION_2	72	17	C3,UCHL1,OAS1,IFI44L,OAS2,CCL5,MMP1,CCL20,XAF1,ICAM1,STAT1,MCAM,IFIT3,DHRS2,TNFSF10,STC1,TNFAIP3	Genes up-regulated in T24 cells (bladder carcinoma) after treatment with decitabine (5-aza-2'-deoxycytidine) [PubChem=451668] .	up	1.00E-04
M13479	TAKEDA_TARGETS_OF_NUP98_HOXA9_FUSION_10D_UP	397	70	RARRES3,CADM1,PTGS2,ISG15,SERPINE2,TPSB2,RAB27B,GATM,SCN2A,LOC25845,IFI44,ZNF503,HS3ST3B1,RTP4,GNAI1,PCDH20,OAS1,OAS2,ARG2,HLA-DPB2,CDC42EP3,BTLA,OASL,IFI27,MIR155HG,FERMT2,ANGPT2,BATF3,TPSAB1,TPSB2,SAMD9L,LY6E,STAT1,LSP1,HPGD,CALB1,CXCL10,DNAJC6,TNFAIP6,	Genes up-regulated in CD34+ [GeneID=947] hematopoetic cells by expression of NUP98-HOXA9 fusion [GeneID=4928;3205] off a retroviral vector at 10 days after transduction.	up	2.00E-04
M6670	TAKEDA_TARGETS_OF_NUP98_HOXA9_FUSION_3D_UP	370	62	ATP1B1,PTGS2,CRHBP,PTGS1,RNF213,CXCL10,ISG15,CH25H,LOX,RAB27B,BST2,DTX3L,CRISP2,SCN2A,ZNF503,SERPING1,IFI44,MECOM,AHR,PARP14,GBP3,GBP1,MVP,IFIH1,OAS1,OAS2,IFI35,CMPK2,HOXA3,HOXA6,HOXA7,HOXA9,S100A10,HOXB3,CDKN1C,IFI27,OASL,PARP9,QPRT,SLC5A3,IFI44L,HEY1,TR	Genes up-regulated in CD34+ [GeneID=947] hematopoetic cells by expression of NUP98-HOXA9 fusion [GeneID=4928;3205] off a retroviral vector at 3 days after transduction.	up	5.00E-04
M7388	TAKEDA_TARGETS_OF_NUP98_HOXA9_FUSION_10D_DN	299	42	SLC7A8,TLR7,C1QC,MMP1,PPP1R1B,TGFBI,MS4A2,GGTA1,OLFML2B,C1QA,C1QB,TACSTD2,CST7,ST14,CLEC5A,CCL4,FOLR3,BLNK,CHI3L1,CCL3,CCL3L1,RIT1,KCNJ15,NRP1,PTGER3,PADI2,MMP12,BPI,PLA2G7,TREM2,HS3ST2,FPR3,RAB42,CD24,GPNMB,PDK4,DACH1,ANXA3,CCL18,RASSF4,TEX15,SLFN13	Genes down-regulated in CD34+ [GeneID=947] hematopoetic cells by expression of NUP98-HOXA9 fusion [GeneID=4928;3205] off a retroviral vector at 10 days after transduction.	up	7.00E-04
M9740	TAKEDA_TARGETS_OF_NUP98_HOXA9_FUSION_8D_DN	416	51	C1QC,PPP1R1B,NCF2,HLA-DQA1,C1QA,C1QB,CLEC5A,SLC11A1,PPP1R3B,CHI3L1,ETS1,CLEC7A,NRP1,SOBP,SERPINA1,KCNMA1,MMP14,MMP12,CD163,PLA2G7,SERPINB1,FPR3,GLIPR2,CD24,PDK4,TEX15,FCGR2B,FCGR2C,CD14,IL10,MMP1,TGFBI,MS4A2,ELOVL7,KRT86,ENC1,PNPLA1,CST7,CLU,ST8SIA1,FOLR3	Genes down-regulated in CD34+ [GeneID=947] hematopoetic cells by expression of NUP98-HOXA9 fusion [GeneID=4928;3205] off a retroviral vector at 8 days after transduction.	up	0
M15057	HENDRICKS_SMARCA4_TARGETS_UP	98	14	TPM1,MMP1,ATP2B4,SERPINE2,AGT,PTPN7,LGALS1,PSG9,CDKN1A,ITGA5,PSG5,PSG4,ARL4C,CTSH	Genes up-regulated in ALAB cells (breast cancer) upon reintroduction of SMARCA4 [GeneID=6597] expressed off adenoviral vector.	up	0.0069
M7914	HENDRICKS_SMARCA4_TARGETS_DN	84	11	NRP2,NRP1,LUM,KCNJ2,CD74,MYC,SFTPB,ARHGDIB,LGALS3,HPGD,HLA-DRA	Genes down-regulated in ALAB cells (breast cancer) upon reintroduction of SMARCA4 [GeneID=6597] expressed off adenoviral vector.	up	0.0128

M1804	WANG_SMARCE1_TARGETS_UP	346	42	CADM1,TGFB1,FAP,GATA3,TGFBI,MATN2,CA12,FRY,GRB10,COL1A1,PMP22,ABCA1,WT1,FN1,PLAT,PLAG1,CHI3L1,KLRC1,KLRC2,HOXB5,HOXB6,NTRK2,MARCKS,PRRX1,BCAM,INHBB,STOM,CA4,VCAN,LUM,COL3A1,DCN,CLEC2B,ACAN,CD24,RNASE4,COL15A1,MAN1C1,TMEM2,NUPR1,SULF1,F2R	Genes up-regulated in BT549 cells (breast cancer) by expression of SMARCE1 [GeneID=6605] off a retroviral vector.	up	0.0248
M4780	WANG_SMARCE1_TARGETS_DN	765	52	PDHA1,APOO,S100A2,PRAME,STC2,CYP1B1,BDKRB1,HSPA1A,CD70,DENND2A,BDKRB2,LIF,TPD52L1,IFI27,LAMA4,BNIP3,TNFRSF11B,IL4R,DLEU2,HSPA1A,HSPA1B,HK2,GNG11,LY6E,TBC1D4,MXRA5,SOD2,TNFRSF6B,DUSP9,SPON2,HADH,EGFR,BST2,HLA-C,HLA-B,CSTB,SLITRK5,PRR7,PSMB8,PSMB9,PLK2,UCHL	Genes down-regulated in BT549 cells (breast cancer) by expression of SMARCE1 [GeneID=6605] off a retroviral vector.	up	0.0019
M4196	NUYTTEN_EZH2_TARGETS_UP	1950	210	LDHA,B2M,TMEM140,ISG20,SERPINE2,ZNF395,RAB27B,NFKBIE,PEA15,AHNAK2,UBE2L6,PARP9,BNIP3,FGF1,PRSS35,ERRFI1,CEACAM1,MMP13,ARL4C,TRIM14,PDK1,TRIM21,TMEM2,ACCN2,CXCL10,TGFBI,IL1B,IL15RA,FNDC3B,HLA-H,HLA-C,HLA-B,HLA-E,HLA-G,HLA-F,VAMP4,IL32,MEIS2,NTN4,CDKN1A,SLC	Genes up-regulated in PC3 cells (prostate cancer) after knockdown of EZH2 [GeneID=2146] by RNAi.	up	0
M4723	TONKS_TARGETS_OF_RUNX1_RUNX1T1_FUSION_HSC_DN	407	59	ADCY7,PDLIM1,CD52,AQP3,KCNK5,SMOX,MGAT1,CD36,PMP22,BIN2,CYP1B1,DUSP10,TYROBP,LGALS1,SASH3,NOD2,PRG2,MYCN,CCND1,SLC27A2,TPCN1,IL10RA,BIRC3,RASSF2,FCGR2A,SLC7A5,GATA2,ANK1,TGFBI,PDXK,CST7,CSTA,ITGAL,LST1,LAPTM5,SLC48A1,RUNX3,TEAD3,TARP,EMR2,NKG7,E2F8,SLA,SL	Genes down-regulated in normal hematopoietic progenitors by RUNX1-RUNX1T1 [GeneID=861;862] fusion.	up	0
M12138	TONKS_TARGETS_OF_RUNX1_RUNX1T1_FUSION_ERYTHROCYTE_UP	331	54	CRHBP,TLR2,TLR7,SLC2A5,MALL,CH25H,FNDC3B,SOCS3,IFI44,SIRPA,LILRB1,COL1A2,VSIG4,CLEC5A,GBP2,NETO2,C3AR1,PALM,ENPP2,OAS2,HOXA9,NDRG1,PLAT,LPL,KLF6,TMEM110,DNASE2,PLK2,QPRT,SLC5A3,UCHL1,ACVR1B,SLC1A3,SERPINA1,LAIR1,PRG2,DHRS9,IFI16,VAT1,CD163,FCGR1A,FCGR1C,V	Genes up-regulated in erythroid lineage cells by RUNX1-RUNX1T1 [GeneID=861;862] fusion .	up	0
M11428	GAURNIER_PSMD4_TARGETS	120	33	HLA-DMB,CXCL11,HLA-DMA,TGFB1,CXCL10,B2M,HLA-H,RELB,HLA-A,HLA-C,HLA-B,HLA-E,HLA-DQA1,HLA-G,HLA-F,CCR5,HLA-DPA1,HLA-DRA,HLA-DQB1,C3AR1,HLA-DQB2,CCL3,HLA-DRB1,C3,CXCL9,CCL5,CCL20,HLA-DRB5,HLA-DPB1,HLA-DPB2,HLA-DOA,CCL18,CXCL16	Inflammatory cytokines	up	0
M14801	HOOI_ST7_TARGETS_DN	257	35	APOBEC3G,MMP1,MCOLN3,VASH1,COL1A1,APOBEC3F,RBP4,LOC400931,PFKFB4,RPS27,PPP1R3B,PPL,OBSL1,NDRG1,ANGPTL4,FN1,MCAM,KDR,CXCL16,PCSK1N,NNMT,SPRY4,CXCR4,MMP13,DHRS2,SRGAP3,PLA2G7,PPFIA4,VIM,FAM46C,ENO2,PDK1,RNASE4,SPARC,SLC14A1	Genes down-regulated in PC-3 cells (prostate cancer) stably expressing ST7 [GeneID=7982] off a plasmid vector.	up	0
M14958	PRAMOONJAGO_SOX4_TARGETS_UP	112	19	BNIP3,MXI1,INSIG2,CXCR4,SLC2A3,IL1B,STC2,GRB10,ADM,VEGFA,STC1,TNFAIP3,TRIB3,GPRC5A,PLIN2,NDRG1,PHLDA1,SOD2,RIT1	Genes up-regulated in ACC3 cells (adenoid cystic carcinoma) after knockdown of SOX4 [GeneID=6659] by RNAi.	up	0
M12391	DAUER_STAT3_TARGETS_UP	78	14	C3,EFNA1,AKAP12,ABCA1,FGG,FGA,SLC2A3,AGT,LBP,SAMD4A,ICAM1,UGCG,SOD2,ADM	Top 50 genes up-regulated in A549 cells (lung cancer) expressing STAT3 [GeneID=6774] off an adenovirus vector.	up	0
M11951	HINATA_NFKB_TARGETS_KERATINOCYTE_UP	144	26	EFNA1,CXCL11,MMP1,CXCL10,IL1B,TNIP1,EGFR,ICAM1,MMP14,TNFAIP6,VEGFA,TNFAIP3,TNFAIP2,GBP1,TNC,VIM,ESM1,CCL5,CYP27B1,CCL20,TAP1,FN1,ARHGDIB,PLAT,SOD2,CDKN1A	Genes up-regulated in primary keratinocytes by expression of p50 (NFKB1) and p65 (RELA) [GeneID=4790;5970] components of NFKB.	up	0
M15491	HAN_SATB1_TARGETS_DN	664	75	PTGS2,OSMR,FSTL1,ISG20,INSIG2,MATN2,CD40,SPAG4,F3,GRAP2,PMP22,GNAI1,ALDOC,OAS1,SERPINH1,LIF,ZAP70,LAMB1,HLA-DOA,PLAT,SYNGR3,APOL2,APOL3,NNMT,BNIP3,P4HA1,ADM,FHL1,TPCN1,BCAS4,TMEM2,QPCT,DUSP1,CDH16,MPZL2,ARNT2,MMP1,SLC16A1,MCOLN3,SLC2A3,TGFBI,IL1B,FNDC3B,P	Genes down-regulated in MDA-MB-231 cells (breast cancer) after knockdown of SATB1 [GeneID=6304] by RNAi.	up	0
M2310	DANG_REGULATED_BY_MYC_DN	488	67	SCPEP1,EDN1,FSTL1,CTGF,PLA1A,CD3E,CRYAB,PRKCDBP,PTPRN,F3,VEGFA,PMP22,ERBB2,AKAP12,SFXN3,OAS1,IFI35,SLC11A1,PEA15,GAS1,JPH2,CANX,DLEU2,MYC,ERRFI1,LYZ,CCND1,ADM,CKAP2L,SERPINB1,COL3A1,GPM6B,MT1A,CHP,DUSP1,FBLN5,WFS1,CSPG4,PMAIP1,TGFB1,CXCL10,SLC2A5,CDKN2B,S	Genes down-regulated by MYC [GeneID=4609]	up	0

M1973	HORIUCHI_WTAP_TARGETS_UP	661	69	RARRES3,MGC21881,OSMR,PTGS1,SERPINE2,RAPGEF5,ERAP1,SMOX,H1F0,STC2,APOLD1,SIPA1L2,ZNF503,IFI44,PITPNM3,TFPI,BTN3A3,PMP22,SLC2A12,EMCN,GCNT2,KCNJ2,PPP1R3C,PCDH17,APOL1,RIT1,HTATIP2,TGFA,CEACAM1,ICAM1,BIRC3,SOD2,QPCT,SELE,TLR3,CXCL11,TAPBP,KLHL6,PAPPA,SLC2A3	Genes up-regulated in primary endothelial cells (HUVEC) after knockdown of WTAP [GeneID=9589] by RNAi.	up	0
M78	EBAUER_TARGETS_OF_PAX3_FOXO1_FUSION_UP	441	43	AQP6,ISG15,CRYAB,CD36,CCR5,EPS8L1,LIF,AHNAK2,KLF6,SCD,TPD52L1,TNNI1,ARHGEF6,SRGAP3,SERPINB1,FHL1,PLEK,GJB1,RASSF4,TUBAL3,ATP6V0A4,TNNC1,LRTM1,CXCL10,SPN,HLA-A,TNS1,COL1A1,TNFAIP2,IL32,GPR37,SST,BCAN,CASQ2,CTF1,ABLIM3,EGLN3,DCN,EHBP1L1,TPM1,HRH1,TGM2,PNOC	Genes up-regulated in Rh4 cells (alveolar rhabdomyosarcoma	up	0
M5672	SU_KIDNEY	32	10	SLC12A1,CUBN,KL,SLC6A13,ENPEP,XPNPEP2,KCNJ1,AQP2,SLC17A1,POU3F4	Genes up-regulated specifically in human kidney tissue.	down	0
M5466	WINTER_HYPOXIA_UP	204	29	LDHA,PGF,PGAM1,BNIP3,SLC16A1,P4HA1,SLC2A1,CA12,CA9,VEGFA,TFAP2C,ALDOA,PFKFB4,IGF2BP2,MIF,PLEKHG3,TPI1,PSMB7,ANKRD37,NDRG1,GAPDH,ANGPTL4,NDUFA4L2,S100A10,HOMER1,SLC6A8,PYGL,PGK1,PLAU	Genes up-regulated in head and neck tumor samples which clustered around known hypoxia genes.	up	0
M14371	NAKAMURA_TUMOR_ZONE_PERIPHERAL_VS_CENTRAL_DN	1349	125	PTGS2,MGC21881,MXI1,RPL10,ZNF395,RAB27B,FAM108C1,UGCG,TFPI,NPHP3,RAC2,ARG2,AHNAK2,OBSL1,GAS1,ACAD11,CACHD1,BNIP3,P4HA1,ST6GALNAC2,SERPINB1,FOXC1,KSR1,C3,HK2,PCDHB14,RPL37,PCDHB10,CCL20,PCSK6,PDK1,SHMT2,TRIM29,TMPRSS4,DUSP1,CP,MOBKL2A,SEZ6L2,UNC5B,SLC2A3,F	Down-regulated genes in peripheral zone of human pancreatic cancer growing in the pancreas of nude mice compared to that of the tumor from the central zone.	up	0
M12225	PICCALUGA_ANGIOIMMUNOBLASTIC_LYMPHOMA_UP	434	81	PGF,FSTL1,C1QC,LIFR,SERPING1,C1QA,C1QB,PMP22,EMCN,CYP1B1,ENPP2,SERPINH1,IDH1,LAMB1,CCDC80,CHI3L1,CD63,LAMA4,IFI27,RGS5,MARCKS,PLA2G4C,NNMT,FERMT2,GJA1,KCNJ10,NR2F2,KCNMA1,PTGDS,C7,C3,COL3A1,CXCL9,SAMD9L,GJC1,CCL21,FLRT2,HSPG2,COL15A1,MXRA5,P2RY13,RASSF4,A	Up-regulated genes in angioimmunoblastic lymphoma (AILT) compared to normal T lymphocytes.	up	0
M12490	ONKEN_UVEAL_MELANOMA_UP	1659	132	PGAM1,B2M,ISG15,HLA-DRA,PEA15,AHNAK2,LAPTM4B,RGS5,HTATIP2,LZTS1,FGF9,LYN,LRP1,SAMD4A,HLA-J,PDXK,HLA-C,HLA-B,HLA-E,HLA-G,HLA-F,IFITM3,MEIS2,CDKN1A,SLC1A4,C4A,CASP1,IFI16,PKM2,CADM1,APOD,PRKCDBP,FRY,C1QA,C1QB,BTN3A3,SFXN3,CD74,IDH2,TYROBP,KDR,ETS2,FKBP11,RI	Genes up-regulated in uveal melanoma: class 2 vs class 1 tumors.	up	0
M14539	BERTUCCI_MEDULLARY_VS_DUCTAL_BREAST_CANCER_UP	442	51	ERAP1,GBP5,BTN3A1,BTN3A3,NFKBIE,IFI30,HLA-DOB,MKI67,RGS1,CLEC7A,MIR155HG,LAG3,RAP2B,ICAM1,GZMA,ANKRD22,CLEC2D,PCSK6,BIRC3,STAT1,SOD2,DRAM1,TLR2,MCM10,IL15RA,SOCS3,DTX3L,RELB,CSDA,GBP4,TNFAIP2,IL32,TAGAP,IL12RB1,STAMBPL1,PSMB8,PSMB9,KLRC1,KLRC2,NBN,HCP5,CT	Genes up-regulated in medullary breast cancer (MBC) relative to ductal breast cancer (DBD).	up	0
M17471	SCHUETZ_BREAST_CANCER_DUCTAL_INVASIVE_UP	739	128	HLA-DMA,HLA-DMB,ADCY7,FSTL1,CD53,SERPINE2,CTGF,FAP,CH25H,OLFML2B,SERPING1,PRKCDBP,HLA-DQA1,C1QA,C1QB,HLA-DPA1,HLA-DRA,IFI30,ESM1,KCNJ2,SERPINH1,CD74,FCER1G,AHNAK2,HLA-DRB6,HLA-DPB1,TCF4,LAMB1,TYROBP,LGALS1,GAS1,KDR,LAMA4,RGS1,SRPX2,CLEC7A,PLAU,NNMT,NRP1,M	Genes up-regulated in invasive ductal carcinoma (IDC) relative to ductal carcinoma in situ (DCIS	up	0
M19875	SENGUPTA_NASOPHARYNGEAL_CARCINOMA_UP	592	73	PTGS2,ZNRF3,CDCA7,ERAP1,EFNB2,UHRF1,ZWINT,ARHGAP8,IDH1,LAMB1,GAD1,CHI3L1,PLAU,NPNT,FERMT1,GJA1,DLEU2,ANGPT2,FCGR3B,TOP2A,KCNMA1,FMNL2,GZMA,GZMB,GZMH,TNFRSF10B,ANKRD22,PLA2G7,VCAN,SLC39A14,COL3A1,CXCL9,FCGR1B,COL15A1,STAT1,FZD7,RASSF4,FCGR2A,DRAM1,CD14,ARN	Genes up-regulated in nsopharyngeal carcinoma relative to the normal tissue.	up	0
M10165	TURASHVILI_BREAST_LOBULAR_CARCINOMA_VS_DUCTAL_NORMAL_UP	136	27	PRRX1,ENPEP,EDNRA,CXCR4,GPX8,LOX,RAP2C,CILP,LYZ,IFI44,PLXDC1,COL1A2,VCAN,COL1A1,COL3A1,TAGAP,HSPA6,WIPF1,LAMB1,THBS2,FN1,SPARC,COL5A2,MXRA5,RGS1,SFRP2,SULF1	Genes up-regulated in lobular carcinoma vs normal ductal breast cells.	up	0
M13547	TURASHVILI_BREAST_LOBULAR_CARCINOMA_VS_LOBULAR_NORMAL_DN	147	23	PRRX1,CXCR4,DLEU2,LOX,UGCG,LYZ,IFI44,COL1A2,VCAN,COL1A1,BTN3A3,LCP2,COL3A1,TAGAP,WIPF1,LAMB1,THBS2,FN1,EPPK1,COL5A2,MXRA5,RGS1,SFRP2	Genes down-regulated in lobular carcinoma vs normal lobular breast cells.	up	0

M4785	FULCHER_INFLAMMATORY_RESPONSE_LECTIN_VS_LPS_DN	1028	127	TMEM140,ISG20,GATM,OLFML2B,SERPING1,SPIRE1,LPCAT1,HIST1H2BK,UBE2L6,APOL3,CD300A,MARCKS,BNIP3,PIK3AP1,PARVG,RAP2B,LYN,SORT1,GNB4,ARL4C,TRIM14,DAPP1,RNASET2,TBC1D2,LILRA6,LILRB3,DUSP1,RNF19B,CXCL11,IL10,CXCL10,TGFBI,IL15RA,FCGR3A,FCGR3B,SAMD4A,TNFAIP6,MNDA,	Genes down-regulated in monocyte-derived dendritic cells (MDDC) after stimulation with galecin-1 (lectin	up	0
M3654	THUM_SYSTOLIC_HEART_FAILURE_UP	908	125	HLA-DMA,HLA-DMB,SERPINE2,SPIRE1,HLA-DPA1,HLA-DRA,NPY6R,PPP1R3C,PPP1R3B,RAC2,ALDH4A1,CDC42EP3,MSR1,HTATIP2,FGF1,ANGPT2,LYN,LYZ,INHBB,CLIC5,VCAN,FPR3,MYOM3,PHLDA1,PDK1,P2RY13,CP,HLA-DRB1,CXCL12,FCGR3A,FCGR3B,CECR1,HLA-C,VSNL1,MNDA,MFAP4,C3AR1,CCL4,ANGPTL1,A	Genes up-regulated in samples with systolic heart failure compared to normal hearts.	up	0
M12570	DEURIG_T_CELL_PROLYMPHOCYTIC_LEUKEMIA_DN	681	77	RARRES3,CD52,GIT2,KLRD1,CRTAM,CD3D,LPAR6,HLA-DPA1,PRDM1,SFXN3,HIP1R,HLA-DPB1,IKZF3,SYNE1,TGFBR3,LEPROTL1,TBX21,RAP2C,GZMA,GZMB,GZMH,GZMK,GNB5,DEGS1,SGCB,PRF1,FHL1,GPR65,GNG11,DAPP1,CLEC2B,BCL2,TBC1D4,KIF21B,MT1H,BIRC3,MT1X,HPGD,KIAA0748,PMAIP1,TCEAL2,APOB	Genes down-regulated in T-PLL cells (T-cell prolymphocytic leukemia) bearing the inv(14)/t(14:14) chromosomal aberration.	up	0
M14507	CHARAFE_BREAST_CANCER_LUMINAL_VS_BASAL_DN	943	106	PTGS2,OSMR,FSTL1,B2M,SERPINE2,FAP,CRYAB,SIRPA,F3,GNAI1,RAC2,CDC42EP3,CCDC80,GAS1,FMNL2,LYN,MMP14,ANXA2P2,C3,FHL1,ITM2C,MT1H,MT1G,PHLDA1,MT1F,TRIM29,AXL,MT1X,PSAT1,TGFBI,FNDC3B,RNF145,EGFR,CSDA,HLA-E,HIF1A,AKR1B10,VSNL1,NFE2L2,TNFAIP3,PTK7,GABRE,CYB5R3,SLC	Genes down-regulated in luminal-like breast cancer cell lines compared to the basal-like ones.	up	0
M9192	CHARAFE_BREAST_CANCER_LUMINAL_VS_MESENCHYMAL_DN	923	91	LDHB,PTGS2,OSMR,FSTL1,SERPINE2,TFPI,PMP22,GNAI1,AHNAK2,AIDA,APCDD1L,KIAA1949,FMNL2,LYN,ANXA2P2,CKAP2L,G0S2,FOXC1,FHL1,GNG11,ITM2C,GLIPR2,BUB1,WIPF1,PHLDA1,FLRT2,AXL,DNM1,TGFB1,SH2D5,CDKN2C,TGFBI,RNF145,EGFR,TNFAIP3,LRRK1,TIMP1,TUBB,CASP1,LOXL1,IFI16,VIM,H	Genes down-regulated in luminal-like breast cancer cell lines compared to the mesenchymal-like ones.	up	0
M17079	VECCHI_GASTRIC_CANCER_ADVANCED_VS_EARLY_UP	342	49	TLR2,FSTL1,SLC2A3,FAP,LOX,STC2,MOXD1,PRELP,TNFAIP6,COL1A2,C3AR1,CYP1B1,APOC1,ESM1,FN1,HAVCR2,LPL,GAS1,SFMBT2,SFRP2,PLAU,SLC5A3,MSR1,PRRX1,EDNRA,SLC2A3,SLC1A3,HEY1,TRIM6,HLA-DQB1,ANGPT2,CD163,PLA2G7,VCAN,GNB4,ARL4C,SNX10,COL3A1,PIK3R5,GPNMB,THBS2,AXL,COL15	Up-regulated genes distinguishing between two subtypes of gastric cancer: advanced (AGC) and early (EGC).	up	0
M986	SMIRNOV_CIRCULATING_ENDOTHELIOCYTES_IN_CANCER_UP	333	55	AQP9,TLR2,CXCL11,SLC7A5,TGFB1,TLR8,APOB,SLC2A3,TGFBI,ZNF395,NCF2,FBP1,TNS1,VEGFA,PMP22,C3AR1,CHKA,CYP1B1,ARHGAP8,CLU,ABCA1,P2RY2,CDA,CHI3L1,MCAM,SYNGR3,ABCG1,CDKN1A,RGS1,ADAP2,EMR2,PLAU,KCNJ15,MARCKSL1,IL21R,SLA,NOD2,CD93,HMOX1,SHC1,IFNGR2,CSF2RA,ABCB1,AD	Genes up-regulated in circulating endothelial cells (CEC) from cancer patients compared to those from healthy donors.	up	0
M6905	JAATINEN_HEMATOPOIETIC_STEM_CELL_DN	498	83	SELENBP1,AQP3,ISG20,S1PR5,NCF2,CD3D,CD36,VSIG1,CX3CR1,OLFM4,CYP1B1,IFI30,ZAP70,FCER1G,TYROBP,LGALS3,CHI3L1,RGS1,ETS1,TGFBR3,CLEC7A,MPEG1,SERPINA1,FCGR3B,GZMA,LYZ,GZMB,GZMH,CD163,GZMK,PLXDC1,VCAN,ARL4C,PRF1,GPR65,FAM46C,IL10RA,QPCT,FCGR2A,CD14,TOB1,SLC2A3,	Genes down-regulated in CD133+ [GeneID=8842] cells (hematopoietic stem cells	up	0
M14427	DODD_NASOPHARYNGEAL_CARCINOMA_DN	2849	183	LDHA,RPLP0,PDHA1,SSTR2,ZWINT,PMP22,SGIP1,PPAPDC1A,AIDA,MMP12,VCAN,DEGS1,FPR3,AXL,PSAT1,CXCL11,CXCL10,IL15RA,FNDC3B,C3AR1,CCL4,RNF165,GLT25D1,KIF23,SLC1A3,UQCRH,SNX10,FKBP5,COL27A1,SCNN1G,XAF1,COL5A2,SCD5,TREX2,IFI44,C1QB,ARHGAP8,PRR5,LMNB1,ARHGAP8,NEK2,EP	Genes down-regulated in nasopharyngeal carcinoma (NPC) compared to the normal tissue.	up	0
M10237	RODRIGUES_THYROID_CARCINOMA_ANAPLASTIC_UP	1487	94	L1CAM,RAB27B,UGCG,SPIRE1,SERPINH1,AHNAK2,CDC42EP3,GAS2L3,ARHGAP11A,RNF175,ANXA2P2,VCAN,ARL4C,HK2,COL7A1,SHMT2,CCNB1,PSAT1,SLC7A5,TGFB1,EIF4EBP1,CDKN2A,SLC2A1,ASPM,EGFR,ENC1,TFAP2A,TNFRSF12A,PLK2,NBN,NXPH4,E2F7,SLFN5,SLC1A4,CASP4,NUSAP1,IFI16,HRH1,XAF1,PLP	Genes up-regulated in anaplastic thyroid carcinoma (ATC) compared to normal thyroid tissue.	up	0
M3645	DELYS_THYROID_CANCER_UP	802	119	FSTL3,FAP,KCNK5,TPSB2,NCF2,C4A,ST14,CYP1B1,ATP11A,MSR1,SERPINA1,FGF1,LYN,INHBB,CCND1,CCND2,G0S2,C3,TRIM14,TRIM29,CCL18,QPCT,SLC7A5,CPA3,STMN2,ENC1,TMSB10,TNFAIP6,MNDA,COL1A1,C3AR1,APOC1,PTK7,MDK,TIMP1,ARHGDIB,MUC1,B3GAT1,CDKN1A,PC,HMOX1,HEY2,PKM2,DDB2,TRE	Genes up-regulated in papillary thyroid carcinoma (PTC) compared to normal tissue.	up	0
M4995	LINDGREN_BLADDER_CANCER_CLUSTER_2B	780	126	ISG20,ACTG2,CTGF,CH25H,FOXF1,CRTAM,C4A,SERPING1,SIRPA,PMP22,HLA-DRA,HLA-DRB1,NFKBIE,RAC2,ZAP70,HLA-DRB5,HLA-DRB6,HLA-DPB1,TCF4,CDC42EP3,CCDC80,RGS5,SERPINA3,PARVG,KIAA1949,LYZ,CPXM1,ARL4C,HLA-DQB1,FHL1,GNG11,GLIPR2,MT1E,MT1H,MT1G,SELL,COL15A1,PDZRN3,MT1X,	Genes specifically up-regulated in Cluster IIb of urothelial cell carcinom (UCC) tumors.	up	0

M15193	GRUETZMANN_PANCREATIC_CANCER_UP	694	86	LDHA,PTGS2,PTGS1,CD53,ISG20,FOXF1,FAP,UGCG,WNK1,PMP22,HLA-DRA,ALDOC,AKAP12,IFI30,OAS1,SERPINH1,PLAT,MKI67,S100A11,S100A10,ABCG1,LAMA4,PLAU,NNMT,P4HA1,IL4R,SERPINA1,FCGR3A,TOP2A,CLIC1,RIN1,VCAN,RAP1GAP,DGKD,THBS2,TRIM29,BIRC3,STAT1,RAB31,YWHAH,PYGL,PGK1,HP	Genes up-regulated in pancreatic ductal adenocarcinoma (PDAC) identified in a meta analysis across four independent studies.	up	0
M16066	SPIELMAN_LYMPHOBLAST_EUROPEAN_VS_ASIAN_DN	1211	61	ISG20,ZNF395,GATM,RAC2,UBE2L6,APOL1,MARCKS,PDIA5,PEPD,ARL4C,RASSF2,SSR2,RBM3,SLC2A5,CECR1,NFE2L2,IL32,ARPC5,GPR27,STK19,TNFSF10,ST8SIA4,NBN,PIP5K1B,PRDX4,RHOB,GPNMB,RNASE6,PNOC,CD53,CD40,LILRB1,LPAR6,OAS2,KLF6,SMAD2,SLAMF7,CD63,SLAMF1,ABCG1,RIT1,ADM,CLEC2	Genes down-regulated in lymphoblastoid cells from the European population compared to those from the Asian population.	up	0
M9585	PUJANA_ATM_PCC_NETWORK	3057	181	KLRC4,LDHB,LDHA,BTK,B2M,SCN2B,OLFML2B,ZWINT,HLA-DRA,RAC2,HLA-DOA,ATP11A,SIRPB1,LYN,ARHGEF18,WAS,PLXDC1,GRIP2,PDK1,TGFB1,CDKN2C,CCL3,ARPC5,CSF2RA,ICOS,ARHGDIB,MUC1,PVT1,FSCN2,P2RX7,SLC1A4,IFI16,CA7,GPSM3,GRAMD4,SCNN1B,HMGN3,CD52,CD53,CD3G,EMX1,IFI44,CD40,B	Genes constituting the ATM-PCC network of transcripts whose expression positively correlated (Pearson correlation coefficient	up	0
M10351	BUYTAERT_PHOTODYNAMIC_THERAPY_STRESS_DN	1337	92	KIFC1,MALL,ISG15,CTGF,HLA-DPA1,PPP1R3C,RAC2,CDC42EP3,MKI67,APOL1,PCDHGA10,INHBB,CCND1,VCAN,TRIM14,HK2,MTCP1NB,RNASET2,BUB1,PCSK6,TBC1D2,SHMT1,MPZL2,CTDSPL,CDKN2C,ST3GAL4,TGFBI,HADH,NEIL3,ENC1,SIX1,NGF,TUBB,MEIS2,ARHGDIB,GINS2,PLK2,LOXL1,SLC43A3,ALDH1B1,NR	Genes down-regulated in T24 (bladder cancer) cells in response to the photodynamic therapy (PDT) stress.	up	0
M12527	RICKMAN_TUMOR_DIFFERENTIATED_WELL_VS_POORLY_DN	775	47	LDHA,ADCY7,PTGS1,LTB4R,ERAP2,S100A2,CD36,PRDM1,GNAI1,AFAP1L1,PVRL4,AHNAK2,SLC31A2,KLF6,CPEB4,CDSN,KCNK10,LOC154761,ALS2CL,ADM,SERPINB1,ARL4D,GPM6B,EPPK1,FEM1C,RAB31,SLC7A5,MMP1,SLC2A1,CA12,TMSB10,ANKRD13A,PTHLH,HIF1A,ERO1L,CDKN1A,YIPF5,CASP4,SQRDL,EGLN3,E	Down-regulated genes that vary between HNSCC (head and neck squamous cell carcinoma) groups formed on the basis of their level of pathological differentiation: well vs poorly differentiated tumors.	up	0
M12369	RICKMAN_METASTASIS_DN	548	69	PTGS2,ADCY7,PGAM1,PDLIM1,MEGF11,ATP2B4,MALL,S1PR5,S100A2,BCR,KIAA1522,PITPNM3,TAGLN2,PVRL4,CD68,ZAP70,FCER1G,AHNAK2,NDUFA4L2,S100A11,SEMA3F,LOC154761,ALS2CL,PRIC285,PRF1,PHLDA3,TBC1D2,EPPK1,TRIM29,RASSF5,PROM2,RNF19B,BCR,MPZL2,MOBKL2A,NLRC5,SLC2A1,LAD1,GN	Genes down-regulated in metastatic vs non-metastatic HNSCC (head and neck squamous cell carcinoma) samples.	up	0
M19826	ZHANG_RESPONSE_TO_IKK_INHIBITOR_AND_TNF_UP	443	62	EFNA1,EDN1,ISG20,LPAR6,NFKBIE,LIF,KLF6,OASL,ETS1,PLAU,MARCKSL1,CHST15,ICAM1,LYN,MMP13,SERPINB9,TNFRSF10B,ANKRD22,G0S2,PRIC285,ABTB2,PML,CCL20,DAPP1,SPSB1,BIRC3,SOD2,RNF19B,DRAM1,OCLN,TLR2,CXCL11,CXCL10,MFHAS1,IL1B,ELOVL7,SAMD4A,SOCS3,RELB,DDIT4,TRIM38,TNF	Genes up-regulated in BxPC3 cells (pancreatic cancer) after treatment with TNF [GeneID=7124] or IKI-1	up	0
M13867	ONDER_CDH1_TARGETS_2_UP	522	60	CADM3,PNMA2,CTGF,STC2,TRPA1,GRB10,TFPI,PMP22,CYP1B1,ENPP2,PPP1R3C,SYNE1,ATP6V0E2,TGFBR3,MARCKS,NNMT,NRP1,FERMT2,KCNMA1,VCAN,COL3A1,GNG11,WIPF1,PTGFR,FBLN1,NUPR1,DUSP1,FBLN5,AOX1,CDKN2C,LOX,ALDH6A1,MOXD1,TNFAIP6,COL1A2,COL1A1,SLITRK5,RHOBTB3,FN1,PODXL,CYFI	Genes up-regulated in HMLE cells (immortalized nontransformed mammary epithelium) after E-cadhedrin (CDH1) [GeneID=999] knockdown by RNAi.	up	0
M15484	ONDER_CDH1_SIGNALING_VIA_CTNNB1	173	29	CADM3,MLPH,EFNA1,FERMT2,PRRX1,KCNMA1,ICAM1,STC2,COL1A2,VCAN,TNFAIP3,ERMP1,IFIH1,C3,SAA2,EPB41L4B,SOX9,TPM1,PPL,NDRG1,SCNN1A,PODXL,MAP1B,FZD3,BIRC3,NUPR1,FBLN5,AOX1,IGFBP3	Genes changed in HMLE cells (mmortalized nontransformed mammary epithelium) after RNAi knockdown of both CDH1 and CTNNB1 [GeneID=999;1499]	up	0
M19467	SANA_TNF_SIGNALING_DN	168	20	CTGF,TPT1,APLN,CDCA7L,EMCN,CLU,TGM2,AHNAK2,MYOF,PHLDA1,ARHGDIB,GIMAP7,DHRS11,ANXA1,NTN4,SMYD2,KDR,VWF,FAM198B,PECAM1	Genes down-regulated in five primary endothelial cell types (lung	up	0
M19695	FLECHNER_BIOPSY_KIDNEY_TRANSPLANT_REJECTED_VS_OK_UP	181	50	CD8A,CD52,CD53,ISG20,SLA,CXCR4,IL4R,FCGR3A,CD3D,GZMA,MOXD1,CD163,HLA-F,C1QB,LILRB4,LCK,UBC,CSTA,VSIG4,GBP2,LST1,ALDOC,APOC1,IFI30,ITGB2,CCL5,RGS10,TNFRSF1B,TYMP,LAPTM5,RAC2,IL10RB,IL10RA,FCGR1A,CD2,FCER1G,INPP5D,GAPDH,TYROBP,PTPRC,HCLS1,UBE2L6,STAT1,PSMB8	Genes up-regulated in kidney biopsies from patients with acute transplant rejection compared to the biopsies from patients with well functioning kidneys more than 1-year post transplant.	up	0

M11620	WIELAND_UP_BY_HBV_INFECTION	240	65	HLA-DMA,HLA-DMB,RARRES3,HLA-DRB1,PTTG1,CD53,CXCL11,CXCL10,ISG20,B2M,TAPBP,CD3D,HLA-A,CECR1,HLA-C,HLA-B,HLA-E,HLA-F,C1QB,HLA-DPA1,VSIG4,GBP2,GBP1,HLA-DRA,C3AR1,IFI30,CCL5,CD74,CD68,LAPTM5,RAC2,FCER1G,HLA-DPB1,ARHGDIB,TYROBP,LGALS3,UBE2L6,S100A10,TARP,PSMB9	Genes induced in the liver during hepatitis B (HBV) viral clearance in chimpanzees.	up	0
M13453	DER_IFN_BETA_RESPONSE_UP	166	26	PMAIP1,ISG15,HADH,PDXK,BST2,HLA-A,HLA-C,IFI16,HLA-E,HIF1A,GBP1,NMI,PML,TRIM14,OAS1,OAS2,IFI35,TAP1,PHLDA1,MAP1B,ARID3A,STAT1,TRIM21,IFIT3,ETS2,IRF1	Genes up-regulated in HT1080 (fibrosarcoma) cells by treatment with interferon beta for 6 h.	up	0
M9826	VANTVEER_BREAST_CANCER_METASTASIS_DN	216	26	SLC2A3,ASPM,PRAME,DEPDC1,VEGFA,CTSL2,ESM1,PAQR3,TK1,LPCAT1,NDRG1,GGH,PLEKHA1,BNIP3,PFKP,NUSAP1,CA9,ADM,DEGS1,NMB,HJURP,BUB1,FLT1,SMC4,CP,PGK1	Genes whose expression is significantly and negatively correlated with poor breast cancer clinical outcome (defined as developing distant metastases in less than 5 years).	up	0
M4551	SANA_RESPONSE_TO_IFNG_UP	138	43	RARRES3,IFI44L,CXCL11,RNF213,HLA-DMA,CXCL10,NLRC5,PLA1A,CASP1,CEACAM1,BST2,DTX3L,HLA-A,SERPING1,HLA-C,HLA-B,HLA-E,HLA-DQA1,PARP14,UBD,HLA-DPA1,GBP3,GBP1,HLA-DQB1,IFIH1,HLA-DRB1,CXCL9,OAS1,OAS2,SAMD9L,IFI35,HLA-DRB5,GIMAP7,SAMHD1,UBE2L6,IDO1,LGALS9,APOL2,A	Genes up-regulated in five primary endothelial cell types (lung	up	0
M17466	SANA_TNF_SIGNALING_UP	152	34	TLR2,IFI44L,CXCL11,TAPBP,CXCL10,CXCR7,RHOB,PLA1A,CASP1,TNIP1,ICAM1,BST2,HLA-A,HLA-C,HLA-B,PARP14,UBD,TNFAIP3,TNFAIP2,GBP1,IFIH1,OAS1,IL32,OAS2,SAMD9L,VCAM1,CCL20,SAMHD1,BIRC3,LGALS9,APOL3,APOL1,SLC15A3,DRAM1	Genes up-regulated in five primary endothelial cell types (lung	up	0
M3069	LENAOUR_DENDRITIC_CELL_MATURATION_DN	202	26	TLR1,ISG15,CCR2,C3AR1,ITGAL,LST1,FCER1G,SREBF2,CORO1A,NAMPT,CYTIP,MARCKSL1,SLC1A3,SERPINA1,LYZ,CD163,BTG2,C3,NMB,TNFRSF1B,CLEC2B,IL2RG,CD180,SOD2,CYBB,CD14	Genes down-regulated during in vitro maturation of CD14+ [GeneID=929] monocytes (day 0) into immature (day 7) and mature dendritic cells (day 14).	up	0
M4861	VERHAAK_AML_WITH_NPM1_MUTATED_UP	416	75	SCPEP1,HIST1H2AD,AQP9,TLR4,CXCL10,KCNK5,RNASE2,LILRA6,IL1B,FCGR3A,FCGR3B,VNN2,FBP1,LILRB1,C1QA,C1QB,LILRB2,CD36,LILRB3,CX3CR1,TNFAIP3,FAM38B,TNFAIP2,MVP,PHKA2,C3AR1,CYP1B1,CLU,IFI30,KCNJ2,CCL4,HOXA6,HOXA7,HOXA9,DEFB1,LAPTM4B,CD300C,HOXB3,TNFSF10,CCL3,CCL3	Genes up-regulated in acute myeloid leukemia (AML) patients with mutated NPM1 [GeneID=4869] .	up	0
M8493	BASSO_CD40_SIGNALING_UP	222	36	HLA-DRB1,BATF,IL1B,NCF2,CD40,HLA-DQA1,HLA-F,DDIT4,TCFL5,TNFAIP3,TNFAIP2,CCL3,NFKBIE,IFI30,CCL4,CD74,LAPTM5,RAC2,HLA-DPB1,LGALS1,NCKAP1L,SLAMF1,RGS1,HLA-DMA,ICAM1,LYN,HLA-DQB1,BCL2,TAP1,SH2B3,CR2,PTPRE,PLEK,TNFSF9,STAT1,HSP90B1	Gene up-regulated by CD40 [GeneID=958] signaling in Ramos cells (EBV negative Burkitt lymphoma).	up	0
M19541	REN_ALVEOLAR_RHABDOMYOSARCOMA_DN	826	73	OSMR,FSTL3,FSTL1,SERPINE2,CTGF,FAP,PEA15,P4HA1,CLIC1,MMP14,CCND1,VCAN,ARL4C,AXL,QPCT,LRP1,DUSP1,TGFB1,TGFBI,FNDC3B,EGFR,PDXK,COL1A1,NGF,TNFRSF12A,ARPC5,TIMP1,VDR,PRDX4,CASP4,LOXL1,VAT1,ANXA1,ANXA5,COL5A2,SCPEP1,SFXN3,TAGLN2,IDH1,PLAT,KLF6,OSBPL3,S100A10,L	Genes commonly down-regulated in human alveolar rhabdomyosarcoma (ARMS) and its mouse model overexpressing PAX3-FOXO1 [GeneID=5077;2308] fusion.	up	0
M8144	KRIGE_RESPONSE_TO_TOSEDOSTAT_6HR_UP	1958	125	SIPA1L2,DUSP10,UBE2L6,ERRFI1,LYZ,SERPINB1,KSR1,PHLDA1,SLFN13,PSAT1,AKNA,HLA-J,HLA-A,HLA-E,HLA-G,HLA-F,VAMP4,TTYH3,PVT1,MYO1G,CDKN1A,E2F7,PREX1,SLC1A4,SPRY1,DDB2,GPSM3,CYBB,ERAP1,GPT2,APOLD1,WNK1,CD40,VEGFA,IDH1,TYROBP,CHI3L1,ETS1,RIT1,NRP1,CPEB2,CPEB4,AGP	Genes up-regulated in HL-60 cells (acute promyelocytic leukemia	up	0
M17915	KRIGE_RESPONSE_TO_TOSEDOSTAT_24HR_UP	1617	120	SIPA1L2,DUSP10,UBE2L6,ERRFI1,SERPINB1,KSR1,PHLDA1,SLFN13,PSAT1,AKNA,HLA-J,HLA-A,HLA-E,HLA-G,HLA-F,VAMP4,TTYH3,MYO1G,CDKN1A,E2F7,PREX1,SLC1A4,IFI16,DDB2,GPSM3,EHBP1L1,CYBB,ERAP1,GPT2,WNK1,APOLD1,CD40,VEGFA,BIN2,IDH1,TYROBP,CHI3L1,ETS1,RIT1,NRP1,CPEB2,CPEB4	Genes up-regulated in HL-60 cells (acute promyelocytic leukemia	up	0

M17363	KIM_LRRC3B_TARGETS	62	20	HLA-DQB1,HLA-DRB1,CXCL9,OAS2,CCL5,CD74,B2M,TAPBP,ISG15,TAP1,SERPINA1,HLA-DPB1,HLA-A,LYZ,HLA-C,IFI44,IFI16,HLA-B,HLA-E,IFI27	Immune response genes up-regulated in zenograft tumors formed by SNU-601 cells (gastric cancer) made to express LRRC3B [GeneID=116135] .	up	0
M3961	KYNG_DNA_DAMAGE_UP	202	35	CRHBP,CD53,SERPINE2,CTGF,LOX,CRYAB,HLA-DQA1,HSPB8,EPS8L1,NGF,GBP1,ITGAL,AKAP12,NDRG1,RPL7A,FN1,NNMT,NRP2,PRDX4,CSF2RB,RHOB,SEMA3B,IFNGR2,CCND1,TNFRSF10B,LCK,VIM,COL3A1,FMO4,IL10RB,MT1H,ANXA1,PCK2,ID1,IGFBP3	Genes with GO annotation and up-regulated after DNA damage in cell lines from young donors.	up	0
M10319	WALLACE_PROSTATE_CANCER_RACE_UP	728	132	ADCY7,CD52,CD53,DNASE1L3,ISG20,ISG15,ERAP1,PDHA1,HCG26,CD3D,APOLD1,OLFML2A,IFI44,PPARGC1A,HLA-DQA1,C1QA,C1QB,LILRB2,BTN3A1,CCR5,VSIG4,BTN3A2,HLA-DRA,OAS2,RAC2,ZAP70,FCER1G,AHNAK2,HLA-DPB1,UBE2L6,NCKAP1L,SLAMF1,IFI27,APOL1,RGS1,HMGCS2,ETS2,EDNRA,HLA-DQB1,G	Genes up-regulated in prostate cancer samples from African-American patients compared to those from the European-American patients.	up	0
M15709	ACEVEDO_LIVER_CANCER_UP	1948	126	B2M,PGAM4,HLA-DRA,SERPINH1,RAC2,GGH,RGS1,RGS5,LYZ,SERPINB1,SORT1,FOXC1,PHLDA3,CXCL10,FNDC3B,HLA-C,HLA-B,HLA-E,HLA-F,TCFL5,NFE2L2,FAM38B,IL32,ARPC5,CCL5,ABCB1,PTTG1IP,SLC1A3,UQCRH,UBD,FKBP5,TMED7,CD2,FRZB,APOLD1,IFI44,PRKCDBP,BTN3A2,CHKA,CD74,TK1,IFI27,ETS	Genes up-regulated in hepatocellular carcinoma (HCC) compared to normal liver samples.	up	0
M2700	SMID_BREAST_CANCER_RELAPSE_IN_BONE_DN	787	91	LDHB,PTGS2,FBXO17,SARS2,EDN1,AQP5,CD52,L1CAM,GLDC,ACTG2,MALL,SOSTDC1,KCNK5,S100A1,S100A2,PRAME,CRYAB,PRELP,VEGFA,VGLL1,TFCP2L1,IGF2BP2,TAGLN2,RAC2,HLA-DOB,KLF6,NDUFA4L2,SFRP1,ELF5,FERMT1,ART3,TNFRSF11B,SCRG1,CEACAM1,MMP14,FOXC1,ARL4C,HLA-DQB2,GPM6B,ITM2C,	Genes down-regulated in bone relapse of breast cancer.	up	0
M17572	SMID_BREAST_CANCER_LUMINAL_B_DN	1353	199	LDHB,PTGS2,L1CAM,ISG20,ACTG2,MALL,SERPINE2,CTGF,PPP1R1A,CH25H,KCNK5,VNN2,CRYAB,SERPING1,VGLL1,TFPI,GRB14,HLA-DRA,EMCN,CYP1B1,SERPINH1,RAC2,ZAP70,HLA-DOB,ELF5,SCRG1,LYN,MICAL3,LYZ,MMP14,MYCN,VCAN,FOXC1,IGF2,INS-IGF2,ARL4C,HLA-DQB1,C7,HSD17B2,C3,FHL1,PML,IT	Genes down-regulated in the luminal B subtype of breast cancer.	up	0
M8513	SMID_BREAST_CANCER_NORMAL_LIKE_UP	1087	153	LDHB,ISG20,BTK,MALL,CH25H,GIT2,VNN2,CRYAB,SERPING1,TFPI,HLA-DPA1,HLA-DRA,EMCN,CYP1B1,RAC2,ZAP70,TCF4,HLA-DOB,SYNE1,RGS5,TGFBR3,LZTS1,ANGPT2,FMNL1,ABCB1,LYN,LYZ,ARL4C,HLA-DQB1,C7,C3,FHL1,GNG11,ITM2C,SORBS1,CCL21,WIPF1,ZBP1,FLRT2,MT1M,SELL,LILRA6,LILRB3,SEL	Genes up-regulated in the normal-like subtype of breast cancer.	up	0
M8124	SMID_BREAST_CANCER_BASAL_UP	1486	177	KIFC1,LDHB,PTGS2,L1CAM,ISG20,ACTG2,MALL,CTGF,KCNK5,CRYAB,VGLL1,RTP4,SERPINH1,HIST1H2BK,MKI67,NDUFA4L2,GGH,ATP11A,FGF9,ELF5,SCRG1,MYC,CEACAM1,LYN,MMP14,MMP12,FOXC1,ARL4C,PML,ITM2C,CCL20,BUB1,SIM1,PHLDA1,PDK1,HAPLN1,TRIM29,MT1X,CCL18,QPCT,CP,PSAT1,DUSP9,MPZ	Genes up-regulated in basal subtype of breast cancer samles.	up	0
M9946	LU_TUMOR_ANGIOGENESIS_UP	52	9	EDN1,TYMP,HEY1,CXCR4,TNFAIP6,CCND2,VEGFA,STC1,VCAN	Up-regulated genes of putative pathways stimulated in tumor endothelial cells by papillary serous ovarian epithelial tumor cells.	up	0
M12636	RUTELLA_RESPONSE_TO_CSF2RB_AND_IL4_UP	691	60	IL6ST,APOO,CYP1B1,LMNB1,SCD,CHI3L1,GGH,SLAMF7,CSRP1,CD63,SLAMF1,ABCG1,SYNGR3,QPRT,MARCKS,NRP1,MARCKSL1,KCNMA1,BATF3,CYB5A,MMP12,ANXA2P2,CCND1,CXCL9,CYP27B1,DAPP1,TBC1D4,BIRC3,PHYH,CCL18,IL10,TBC1D13,TNFAIP6,LRRK1,NETO2,APOC1,ST8SIA1,VDR,NDRG1,ARL6IP5,RUNX	Genes up-regulated in peripheral blood monocytes by CSF2RB (GM-CSF) and IL4 [GeneID=1437;3565].	up	0
M8582	RUTELLA_RESPONSE_TO_CSF2RB_AND_IL4_DN	674	85	RARRES3,AQP9,CD52,LTB4R,ZNF395,CRTAM,HLA-DQA1,C1QA,LILRB2,CCR5,CX3CR1,LILRB4,VSIG4,CLEC5A,CD68,LGALS3,ACPP,CD300A,ETS2,PDIA5,CHST15,BHLHE41,ARHGEF6,SIGIRR,CD163,PTGDS,PLA2G7,VCAN,C3,HK2,PLIN2,RNASET2,MT1H,MT1G,MT1F,BTN3A2,BTN3A3,SELL,STAT1,PCK2,MT1X,SOD2,	Genes down-regulated in peripheral blood monocytes by CSF2RB (GM-CSF) and IL4 [GeneID=1437;3565].	up	0

M18149	RUTELLA_RESPONSE_TO_HGF_UP	874	98	CRTAM,CYP1B1,GGH,SYNGR3,MARCKS,BHLHE41,MMP12,ANXA2P2,CCND1,C3,HK2,TBC1D4,MT1H,MT1G,MT1F,PCK2,PHYH,MT1X,CCL18,IL10,CXCL10,MFHAS1,IL1B,PDXK,FBP1,TNFAIP6,NETO2,C3AR1,APOC1,ST8SIA1,VDR,ADAM28,NKG7,CCRL2,SLC1A4,SLC1A3,CSF2RA,SLC43A3,VAT1,FCGR1A,FCGR1C,SNX10,GP	Genes up-regulated in peripheral blood monocytes by HGF [GeneID=3082] .	up	0
M6413	RUTELLA_RESPONSE_TO_HGF_DN	491	47	RARRES3,CD52,LTB4R,ZNF395,HLA-DQA1,LILRB2,CX3CR1,LMNB1,ACPP,CD300A,ETS2,QPRT,MARCKSL1,PDIA5,BATF3,ARHGEF6,CD163,SIGIRR,VCAN,DAPP1,PLIN2,RNASET2,SELL,SLC16A5,CD14,SLC2A3,HLA-E,DDIT4,LRRK1,ITGAL,MCL1,RUNX3,ITGA4,CDKN1C,CDKN1A,NAMPT,SLC2A3,CXCR4,CASP1,NLRP3,	Genes down-regulated in peripheral blood monocytes by HGF [GeneID=3082] .	up	0
M19971	RUTELLA_RESPONSE_TO_HGF_VS_CSF2RB_AND_IL4_UP	858	102	ZNF395,CRTAM,HLA-DQA1,CYP1B1,GGH,ACPP,CD300A,PDIA5,BHLHE41,CCND1,C3,HK2,RNASET2,MT1H,MT1G,MT1F,SELL,PCK2,MT1X,CCL18,IL10,CXCL10,MFHAS1,SLC2A3,IL1B,PDXK,FBP1,HLA-E,DDIT4,TNFAIP6,C3AR1,APOC1,ADAM28,CDKN1C,NKG7,CCRL2,SLC1A4,SLC1A3,CASP1,SLC43A3,VAT1,FCGR1A,F	Genes up-regulated in peripheral blood mononucleocytes by HGF [GeneID=3082] compared to those regulated by CSF2RB (GM-CSF) and IL4 [GeneID=1437;3565].	up	0
M13333	RUTELLA_RESPONSE_TO_HGF_VS_CSF2RB_AND_IL4_DN	507	43	IL6ST,LTB4R,LMNB1,CSRP1,SLAMF1,SYNGR3,ABCG1,MARCKS,QPRT,MARCKSL1,BATF3,ARHGEF6,CYB5A,MMP12,ANXA2P2,VCAN,DAPP1,TBC1D4,BIRC3,PHYH,SLC16A5,TBC1D13,LRRK1,NETO2,MCL1,ST8SIA1,VDR,ARL6IP5,RUNX3,ITGA4,CDKN1A,IL21R,CXCR4,RAMP1,TUBA1C,CSF2RA,CD84,GABBR1,UBD,EGLN3,M	Genes down-regulated in peripheral blood mononucleocytes by HGF [GeneID=3082] compared to those regulated by CSF2RB (GM-CSF) and IL4 [GeneID=1437;3565].	up	0
M8520	SHEDDEN_LUNG_CANCER_POOR_SURVIVAL_A6	938	75	LDHB,SPAG5,ZWINT,ESM1,MKI67,GGH,ANGPT2,MMP12,ARL4D,HK2,BUB1,CCNB1,DUSP9,PSAT1,SLC7A5,EIF4EBP1,CDKN2A,CDKN2C,SLC2A1,ASPM,NEIL3,NETO2,GINS2,GPR37,KIF23,NBN,E2F8,PAX8,NUSAP1,EGLN3,PTTG1,PITX1,STC2,DEPDC1,VEGFA,STC1,CTSL2,LMNB1,NEK2,CD70,TK1,SCD,RAD54L,TOP2A,	Cluster 6 of method A: up-regulation of these genes in patients with non-small cell lung cancer (NSCLC) predicts poor survival outcome.	up	0
M11197	HSIAO_HOUSEKEEPING_GENES	851	71	RPL17,LDHB,LDHA,PGAM1,ISG20,B2M,RPLP0,RPL12,RPS19,RPS14,HLA-DPA1,HLA-DRA,HLA-DOA,RPS8,UBA1,MARCKS,SERPINA3,CLIC1,RPL41,CCND2,HLA-DQB1,FHL1,RPL35,RPL39,DUSP1,SSR2,HLA-A,TMSB10,HLA-E,TUBB,RPL7A,ARHGDIB,CYB5R3,HINT1,RPS2,UQCRH,HLA-A,HLA-B,UBC,ABLIM1,VIM,PLP2	Housekeeping genes identified as expressed across 19 normal tissues.	up	0
M215	LIU_VAV3_PROSTATE_CARCINOGENESIS_UP	180	38	TLR2,APOD,CDKN2C,SERPINA3,CASP1,PITX2,CTSS,C1QA,CD36,CCND2,LILRB3,PLA2G7,SERPINB1,GBP2,C3,TNC,ITGB2,CD74,TAP1,TGM2,SFTPD,FCER1G,GPNMB,ARHGDIB,SIM2,FN1,TYROBP,PLAT,LGALS3,KLK3,STAT1,PSMB8,PSMB9,IFIT3,CYBB,TNFSF10,FBLN1,IRF1	Selected genes up-regulated in prostate tumors developed by transgenic mice overexpressing VAV3 [GeneID=10451] in prostate epithelium.	up	0
M8467	JISON_SICKLE_CELL_DISEASE_UP	384	54	ATP1B1,CXCL10,HIST1H2BK,SLC2A3,SMOX,H1F0,BST2,HLA-A,CHP2,IFI44,SERPING1,CCR2,VSIG4,GBP2,GBP1,LMNB1,NFKBIE,IFI30,IFI35,TIMP1,FN1,P4HB,TP53I11,UBE2L6,PSMB8,PSMB9,OASL,CDKN1A,RIT1,HTATIP2,PRDX4,CCRL2,HMOX1,CLIC1,ADM,NGFR,HLA-DQB1,ABLIM3,RSAD2,GNG11,TPM1,TYMP	Genes up-regulated in peripheral blood mononuclear cells (PBMC) from sickle cell disease patients compared to those from healthy subjects.	up	0
M5369	POOLA_INVASIVE_BREAST_CANCER_UP	701	116	ADCY7,CD52,PTTG1,CD53,BTK,ISG20,FAP,CH25H,NCF2,CD3D,CD40,HLA-DQA1,LILRB1,LILRB2,LILRB4,NEK2,IFI30,CD74,TK1,CD68,FCER1G,HLA-DRB6,IKZF1,LGALS9,CLEC7A,TOP2A,FCGR3B,GZMA,LYZ,GZMB,MMP13,MMP12,CD163,GZMK,PLA2G7,VCAN,FKBP11,GPR65,CXCL9,FPR3,FCGR1B,PLEK,EPPK1,SEL	Genes up-regulated in atypical ductal hyperplastic tissues from patients with (ADHC) breast cancer vs those without the cancer (ADH).	up	0
M12602	WOO_LIVER_CANCER_RECURRENCE_UP	215	33	TGFB1,CTGF,LOX,CRYAB,TMSB10,TACSTD2,COL1A2,COL1A1,PMP22,FGFR2,ALDOA,ENPP2,SOX9,LPCAT1,SEMA6A,LAMA4,RGS1,MARCKS,PLAU,BHLHE41,PFKP,CCND2,PKM2,TREM2,SGCB,HK2,RGS10,COL9A2,BCL2,ENO2,CD24,DPYSL2,IGFBP3	Genes positively correlated with recurrence free survival in patients with hepatitis B-related (HBV) hepatocellular carcinoma (HCC).	up	0

M5311	HOSHIDA_LIVER_CANCER_SUBCLASS_S1	476	73	LDHB,CD53,CTGF,CD3D,HLA-DQA2,C1QB,ALDOA,CYP1B1,IFI30,CD74,PEA15,HLA-DPB1,TCF4,HLA-DOA,LAMB1,LGALS1,S100A11,S100A10,SMAD2,LGALS9,RIT1,FCGBP,LYN,CCND2,VCAN,HLA-DQB1,COL3A1,HCLS1,COL15A1,LSP1,RAB31,FCGR2A,PGK1,CRABP2,PKMYT1,SLC7A5,PNP,SLC2A5,SLC2A1,IL15RA,LO	Genes from 'subtype S1' signature of hepatocellular carcinoma (HCC): aberrant activation of the WNT signaling pathway.	up	0
M17951	NAKAYAMA_SOFT_TISSUE_TUMORS_PCA1_UP	221	50	IFI44L,CD52,CXCL10,ISG20,SLA,IL4R,FAP,SERPINA1,FCGR3A,FCGR3B,HLA-DQB1,HLA-DQA1,GZMA,LYZ,CD163,C1QA,LILRB1,TNFAIP6,C1QB,SIGLEC1,GZMK,FCGR1A,FCGR1C,HLA-DPA1,VSIG4,BIN2,HLA-DRA,GABBR1,UBD,HLA-DQB1,C3,LUM,CXCL9,IFI30,DCN,CCL5,TIMP1,HSPA6,GPNMB,THBS2,MS4A4A,NC	Top 100 probe sets contrubuting to the positive side of the 1st principal component; predominantly associated with spindle cell and pleomorphic sarcoma samples.	up	0
M9893	RODWELL_AGING_KIDNEY_NO_BLOOD_UP	339	53	OCLN,VTCN1,SERPINE2,RPL12,TPSB2,SPARCL1,SERPING1,CDO1,MOXD1,C1QA,C1QB,VSNL1,COL1A2,TFPI,COL1A1,SERPINH1,TIMP1,FN1,P4HB,MCAM,RAD54L,RGS1,PLK2,NNMT,MSR1,TNFRSF11B,SLC1A3,C4A,BHLHE41,LOXL1,AMBP,DEGS1,GABBR1,UBD,C7,C3,LUM,FHL1,TNC,COL3A1,RPL35,TPM1,ITM2C,VCAM	Genes whose expression increases with age in normal kidney	up	0
M5389	RODWELL_AGING_KIDNEY_UP	807	139	HLA-DMA,HLA-DMB,RARRES3,VTCN1,CD53,MALL,SERPINE2,RPL10,RPL12,TPSB2,SERPING1,VASH1,MGAT1,C1QA,C1QB,RPS19,CX3CR1,TFPI,HLA-DPA1,HLA-DRA,TAGLN2,LPCAT1,RAC2,FCER1G,HLA-DRB6,TYROBP,IKZF1,MCAM,RAD54L,RGS1,CLEC7A,NNMT,HTATIP2,MSR1,TNFRSF11B,CHST15,BHLHE41,HLA-DQB	Genes whose expression increases with age in normal kidney.	up	0
M1278	MCLACHLAN_DENTAL_CARIES_DN	610	119	HLA-DMA,HLA-DMB,PTGS2,CD53,AQP3,SERPINE2,NCF2,KIF5C,HLA-DQA1,C1QB,CCR2,HLA-DPA1,HLA-DRA,XIST,SAA2,IFI30,CD74,RAC2,FCER1G,HLA-DRB6,HLA-DPB1,BLNK,CHI3L1,RAB7L1,CLEC7A,SASH3,PLAU,MSR1,SERPINA3,SERPINA1,HLA-DQB1,FCGR3B,ICAM1,LYN,LYZ,GZMB,ADM,PLA2G7,G0S2,FKBP1	Genes down-regulated in pulpal tissue extracted from carious teeth.	up	0
M10454	MCLACHLAN_DENTAL_CARIES_UP	554	111	HLA-DMA,HLA-DMB,PTGS2,CD53,AQP3,SERPINE2,NCF2,HLA-DQA1,C1QB,CCR2,HLA-DPA1,HLA-DRA,SAA2,IFI30,CD74,RAC2,FCER1G,HLA-DRB6,HLA-DPB1,BLNK,CHI3L1,RAB7L1,CLEC7A,PLAU,SASH3,MSR1,SERPINA3,SERPINA1,HLA-DQB1,FCGR3B,ICAM1,LYN,LYZ,GZMB,ADM,PLA2G7,G0S2,FKBP11,CHL1,HLA-	Genes up-regulated in pulpal tissue extracted from carious teeth.	up	0
M12299	SEMENZA_HIF1_TARGETS	70	25	ALDOA,LDHA,ALDOC,EDN1,HK2,BNIP3,TPI1,SLC2A3,HMOX1,SLC2A1,GAPDH,EPO,ENO1,FLT1,AK3,IGF2,CDKN1A,CA9,ADM,PKM2,ADRA1B,CP,IGFBP2,PGK1,IGFBP3	Genes that are transcriptionally regulated by HIF1A [GeneID=3091] .	up	0
M9221	BROWNE_INTERFERON_RESPONSIVE_GENES	138	39	RARRES3,TLR3,APOBEC3G,IFI44L,CXCL11,ISG20,CXCL10,ISG15,IL15RA,CASP1,BST2,KIF5C,IFI44,HLA-F,GBP2,GBP1,NMI,TRIM14,PML,RSAD2,IFI30,OAS1,OAS2,IFI35,TYMP,TAP1,XAF1,SAMHD1,UBE2L6,IDO1,STAT1,PSMB8,TRIM21,PSMB9,IFIT3,IFIT2,IFI27,OASL,TNFSF10	Genes up-regulated in primary fibroblast culture after treatment with interferon alpha for 6 h.	up	0
M9809	KEGG_CYTOKINE_CYTOKINE_RECEPTOR_INTERACTION	536	70	IL6ST,OSMR,LIFR,CD40,CCR5,CCR2,CX3CR1,VEGFA,TNFRSF6B,CD70,LIF,KDR,BMP7,TNFRSF11B,CCL3L1,IL4R,CCL3L3,INHBB,TNFRSF10B,IL12B,CXCL9,CCL20,IL10RB,CCL21,IL10RA,CCL18,TNFSF14,CXCL11,CXCL12,IL10,TGFB1,CXCL10,IL1B,CSF3R,IL15RA,EGFR,CCL4L2,RELT,CCL3,IL22RA1,TNFRSF1	Cytokine-cytokine receptor interaction	up	0
M16476	KEGG_CELL_ADHESION_MOLECULES_CAMS	270	51	CADM3,OCLN,CADM1,L1CAM,SPN,HLA-A,HLA-C,CD40,HLA-B,HLA-E,HLA-DQA2,HLA-G,HLA-DQA1,HLA-F,HLA-DPA1,HLA-DRA,ITGAL,HLA-DRB1,ITGB2,ICOS,HLA-DRB5,HLA-DPB1,HLA-DOA,HLA-DOB,CD276,ITGA4,CLDN8,CD8A,CD8B,HLA-DMB,HLA-DMA,PDCD1,SDC3,ICAM1,SIGLEC1,VCAN,HLA-DQB1,CLDN16,CL	Cell adhesion molecules (CAMs)	up	0

M16004	KEGG_ANTIGEN_PROCESSING_AND_PRESENTATION	180	41	KLRC4,KLRC2,KLRC3,PDIA3,CD8A,CD8B,HLA-DMB,CANX,HLA-DMA,B2M,TAPBP,KLRD1,CIITA,HLA-A,HLA-C,HLA-B,CTSS,HLA-E,HLA-DQA2,HLA-DQA1,HLA-G,HLA-F,HLA-DPA1,KLRC1,HLA-DRA,HLA-DQB1,HLA-DRB1,IFI30,HSPA1A,HSPA1B,CD74,HSPA2,TAP2,TAP1,HSPA6,HLA-DRB5,CD4,HLA-DPB1,HSPA5,HLA	Antigen processing and presentation	up	0
M5669	KEGG_NATURAL_KILLER_CELL_MEDIATED_CYTOTOXICITY	276	37	KLRC2,KLRC3,KLRD1,CHP2,HLA-A,HLA-C,HLA-B,HLA-E,HLA-G,HCST,KLRC1,ITGAL,KLRK1,ITGB2,RAC2,ZAP70,FCER1G,TYROBP,VAV1,TNFSF10,FASLG,SHC1,FCGR3A,IFNGR2,FCGR3B,ICAM1,GZMB,IFNAR2,TNFRSF10B,LCK,LCP2,PRF1,CD244,CD247,PIK3R5,CHP,SH3BP2	Natural killer cell mediated cytotoxicity	up	0
M615	KEGG_INTESTINAL_IMMUNE_NETWORK_FOR_IGA_PRODUCTION	98	21	HLA-DQB1,HLA-DRB1,HLA-DMB,CXCL12,HLA-DMA,IL10,TGFB1,CXCR4,ICOS,IL15RA,HLA-DRB5,HLA-DPB1,HLA-DOA,HLA-DOB,CD40,PIGR,ITGA4,HLA-DQA2,HLA-DQA1,HLA-DPA1,HLA-DRA	Intestinal immune network for IgA production	up	0
M12617	KEGG_TYPE_I_DIABETES_MELLITUS	90	25	HLA-DQB1,PRF1,HLA-DRB1,FASLG,HLA-DMB,HLA-DMA,HLA-DRB5,IL1B,HLA-DPB1,HLA-DOA,GAD1,HLA-DOB,HLA-A,GZMB,HLA-C,HLA-B,PTPRN,HLA-E,HLA-DQA2,HLA-G,HLA-DQA1,HLA-F,HLA-DPA1,IL12B,HLA-DRA	Type I diabetes mellitus	up	0
M3126	KEGG_LEISHMANIA_INFECTION	146	31	PTGS2,MARCKSL1,TLR2,TLR4,HLA-DMB,HLA-DMA,TGFB1,IL10,IL1B,FCGR3A,FCGR3B,IFNGR2,NCF2,HLA-DQA2,HLA-DQA1,HLA-DPA1,IL12B,HLA-DRA,HLA-DQB1,HLA-DRB1,C3,ITGB2,FCGR1A,HLA-DRB5,HLA-DPB1,HLA-DOA,HLA-DOB,ITGA4,STAT1,FCGR2C,FCGR2A	Leishmania infection	up	0
M13950	KEGG_ASTHMA	62	17	HLA-DQB1,HLA-DRB1,HLA-DMB,HLA-DMA,IL10,MS4A2,FCER1G,HLA-DRB5,HLA-DPB1,HLA-DOA,HLA-DOB,PRG2,CD40,HLA-DQA2,HLA-DQA1,HLA-DPA1,HLA-DRA	Asthma	up	0
M13103	KEGG_AUTOIMMUNE_THYROID_DISEASE	108	23	FASLG,HLA-DMB,HLA-DMA,IL10,HLA-A,HLA-C,GZMB,HLA-B,CD40,HLA-E,HLA-DQA2,HLA-G,HLA-DQA1,HLA-F,HLA-DPA1,HLA-DRA,HLA-DQB1,PRF1,HLA-DRB1,HLA-DRB5,HLA-DPB1,HLA-DOA,HLA-DOB	Autoimmune thyroid disease	up	0
M4741	KEGG_SYSTEMIC_LUPUS_ERYTHEMATOSUS	282	34	C1QC,IL10,HIST1H2BK,C4A,CD40,HLA-DQA2,HLA-DQA1,C1QA,C1QB,HLA-DPA1,HLA-DRA,HLA-DRB1,HLA-DRB5,HLA-DPB1,HLA-DOA,HLA-DOB,HIST1H3D,HIST1H3H,HLA-DMB,HLA-DMA,FCGR3A,FCGR3B,HLA-DQB1,C7,C3,HIST1H2AD,FCGR1A,HIST1H2BE,TRIM21,FCGR2B,FCGR2C,H2AFY2,HIST1H2AJ,FCGR2A	Systemic lupus erythematosus	up	0
M18615	KEGG_ALLOGRAFT_REJECTION	78	24	HLA-DQB1,PRF1,HLA-DRB1,FASLG,HLA-DMB,HLA-DMA,IL10,HLA-DRB5,HLA-DPB1,HLA-DOA,HLA-DOB,HLA-A,GZMB,HLA-C,HLA-B,CD40,HLA-E,HLA-DQA2,HLA-G,HLA-DQA1,HLA-F,HLA-DPA1,IL12B,HLA-DRA	Allograft rejection	up	0
M13519	KEGG_GRAFT_VERSUS_HOST_DISEASE	86	24	HLA-DQB1,PRF1,HLA-DRB1,FASLG,HLA-DMB,HLA-DMA,HLA-DRB5,IL1B,HLA-DPB1,HLA-DOA,KLRD1,HLA-DOB,HLA-A,GZMB,HLA-C,HLA-B,HLA-E,HLA-DQA2,HLA-G,HLA-DQA1,HLA-F,HLA-DPA1,KLRC1,HLA-DRA	Graft-versus-host disease	up	0
M12294	KEGG_VIRAL_MYOCARDITIS	148	28	MYH15,HLA-DMB,HLA-DMA,ICAM1,HLA-A,HLA-C,CD40,HLA-B,HLA-E,HLA-DQA2,HLA-DQA1,HLA-G,HLA-F,CCND1,HLA-DPA1,HLA-DRA,SGCB,HLA-DQB1,ITGAL,PRF1,HLA-DRB1,DAG1,ITGB2,RAC2,HLA-DRB5,HLA-DPB1,HLA-DOA,HLA-DOB	Viral myocarditis	up	0
M7146	BIOCARTA_CLASSIC_PATHWAY	30	6	C7,C4A,C3,C1QC,C1QA,C1QB	Classical Complement Pathway	up	0
M917	BIOCARTA_COMP_PATHWAY	40	7	C7,C4A,MASP1,C3,C1QC,C1QA,C1QB	Complement Pathway	up	0
M19752	REACTOME_COMPLEMENT_CASCADE	102	7	MASP1,C1QC,C4A,C1QA,C1QB,C7,C3	Genes involved in Complement cascade	up	0
M17386	REACTOME_COSTIMULATION_BY_THE_CD28_FAMILY	142	25	HLA-DMB,HLA-DMA,PDCD1,CD3G,LYN,CD3D,CD3E,HLA-DQA2,HLA-DQA1,LCK,HLA-DPA1,GRAP2,HLA-DRA,HLA-DQB1,HLA-DRB1,CD247,TRIB3,ICOS,HLA-DRB5,CD4,HLA-DPB1,HLA-DOA,HLA-DOB,VAV1,BTLA	Genes involved in Costimulation by the CD28 family	up	0
M13166	REACTOME_DOWNSTREAM_TCR_SIGNALING	96	20	HLA-DQB1,HLA-DRB1,CD247,HLA-DMB,HLA-DMA,HLA-DRB5,CD4,INPP5D,HLA-DPB1,HLA-DOA,HLA-DOB,CD3G,CD3D,CD3E,HLA-DQA2,HLA-DQA1,CARD11,LCK,HLA-DPA1,HLA-DRA	Genes involved in Downstream TCR signaling	up	0

M16523	REACTOME_GENERATION_OF_SECOND_MESSENGER_MOLECULES	76	23	HLA-DQB1,HLA-DRB1,CD247,HLA-DMB,HLA-DMA,ZAP70,HLA-DRB5,CD4,HLA-DPB1,HLA-DOA,HLA-DOB,ITK,CD3G,CD3D,CD3E,WAS,HLA-DQA2,HLA-DQA1,LCK,HLA-DPA1,GRAP2,HLA-DRA,LCP2	Genes involved in Generation of second messenger molecules	up	0
M8240	REACTOME_IMMUNOREGULATORY_INTERACTIONS_BETWEEN_A_LYMPHOID_AND_A_NON_LYMPHOID_CELL	214	34	B2M,KLRD1,CRTAM,CD3G,CD3D,CD3E,HLA-A,HLA-C,CD40,HLA-B,HLA-E,HLA-G,HLA-F,HCST,LILRB1,LILRB2,LILRB5,LILRB3,LILRB4,KLRC1,ITGAL,KLRK1,ITGB2,TYROBP,ITGA4,CD8A,CD8B,FCGR3A,ICAM1,C3,CD247,VCAM1,SELL,FCGR2B	Genes involved in Immunoregulatory interactions between a Lymphoid and a non-Lymphoid cell	up	0
M6121	REACTOME_INITIAL_TRIGGERING_OF_COMPLEMENT	88	6	MASP1,C3,C1QC,C4A,C1QA,C1QB	Genes involved in Initial triggering of complement	up	0
M18810	REACTOME_PD1_SIGNALING	60	19	HLA-DQB1,HLA-DRB1,CD247,HLA-DMB,HLA-DMA,PDCD1,HLA-DRB5,CD4,HLA-DPB1,HLA-DOA,HLA-DOB,CD3G,CD3D,CD3E,HLA-DQA2,HLA-DQA1,LCK,HLA-DPA1,HLA-DRA	Genes involved in PD-1 signaling	up	0
M12494	REACTOME_PHOSPHORYLATION_OF_CD3_AND_TCR_ZETA_CHAINS	54	19	HLA-DQB1,HLA-DRB1,CD247,HLA-DMB,HLA-DMA,HLA-DRB5,CD4,HLA-DPB1,HLA-DOA,HLA-DOB,PTPRC,CD3G,CD3D,CD3E,HLA-DQA2,HLA-DQA1,LCK,HLA-DPA1,HLA-DRA	Genes involved in Phosphorylation of CD3 and TCR zeta chains	up	0
M15381	REACTOME_TCR_SIGNALING	130	26	HLA-DMB,HLA-DMA,CD3G,CD3D,CD3E,WAS,HLA-DQA2,HLA-DQA1,CARD11,LCK,HLA-DPA1,GRAP2,HLA-DRA,LCP2,HLA-DQB1,HLA-DRB1,CD247,ZAP70,HLA-DRB5,CD4,INPP5D,HLA-DPB1,HLA-DOA,HLA-DOB,PTPRC,ITK	Genes involved in TCR signaling	up	0
M17299	CHARAFE_BREAST_CANCER_LUMINAL_VS_MESENCHYMAL_UP	942	94	SELENBP1,RAB27B,TOX3,KIAA1522,ST14,ABAT,ERBB2,DAAM1,HIP1R,PVRL4,SLC29A2,CASZ1,ALDH4A1,TMEM191A,ST6GALNAC2,INHBB,DOK7,ESRP1,ESRP2,DEGS2,EPB41L4A,TMPRSS2,EPPK1,PCK2,SLC7A8,AZGP1,LAD1,FBP1,GRHL1,OVOL2,MUC1,FREM2,PREX1,ANO1,SYNGR1,RHOB,EPN3,PADI2,BSPRY,GRAMD4	Genes up-regulated in luminal-like breast cancer cell lines compared to the mesenchymal-like ones.	down	0
M12795	CHARAFE_BREAST_CANCER_BASAL_VS_MESENCHYMAL_UP	254	35	RAB25,LAD1,KIAA1522,TACSTD2,AKR1B10,ST14,OVOL2,PPL,BLNK,MUC1,CBLC,TMEM191A,STEAP4,DMKN,SERPINA3,PPP1R14C,EPN3,BSPRY,TLCD1,ESRP1,ESRP2,EPB41L4A,EHF,GRAMD2,PRSS8,TSC22D3,MARVELD2,CD24,SCNN1A,KLK6,TRIM29,ATP1A1,RNF43,PROM2,GPR110	Genes up-regulated in basal-like breast cancer cell lines as compared to the mesenchymal-like ones.	down	0
M14791	SABATES_COLORECTAL_ADENOMA_DN	590	80	GLDN,L1CAM,HOXD10,TMEM140,GUCA2B,KIF5C,LOC25845,LIFR,CD36,CHGB,GCNT2,MST1,AKAP12,CDA,WSCD1,NPY,SFRP1,SLC26A2,CHRDL1,C7,ALPI,HSD17B2,USP2,MFSD4,GPM6B,CCL21,FLRT2,HAPLN1,MT1M,LRRN2,ATP1A2,PCK1,FBLN1,DUSP1,CP,NR5A2,ADH1B,CXCL12,TLR7,CHAD,CDKN2B,ANK2,MS4A1,CH	Genes down-regulated in colorectal adenoma compared to normal mucosa samples.	down	0
M19062	DODD_NASOPHARYNGEAL_CARCINOMA_UP	3823	246	BTC,ZNF395,GATM,UGCG,DNAH11,DUSP26,DMKN,BHLHE41,ALS2CL,RNF175,KNDC1,TRIM29,DUSP1,GLYATL1,SPATA18,NFE2L2,CDKN1C,EDAR,LYPD6B,ADH6,LRG1,SCNN1B,SCNN1A,FLJ23867,PRR15,CADM4,DNAJB13,SAA2,CD74,CNKSR1,SRPX2,STEAP4,SLC26A2,SIGIRR,PTGDS,CD180,GJB1,SLC16A5,SLC16A9,A	Genes up-regulated in nasopharyngeal carcinoma (NPC) compared to the normal tissue.	down	0
M13273	DELYS_THYROID_CANCER_DN	430	74	GCAT,DNASE1L3,GLDC,APOD,WWOX,MATN2,CRYAB,LIFR,MAGED4,CD36,TFPI,GRB14,EMCN,GNAI1,AKAP12,ALDH4A1,ESRRG,GAS1,SYNE1,SFRP1,TGFBR3,FCGBP,TNFRSF11B,CLMN,MYCN,CHRDL1,PLA2G7,FHL1,GNG11,EPB41L4B,CCL21,BCL2,MT1E,TBC1D4,MT1H,MT1G,MT1F,FLRT2,IGF2,PHYH,MT1X,FBLN1,CDH16	Genes down-regulated in papillary thyroid carcinoma (PTC) compared to normal tissue.	down	0
M1475	FLECHNER_BIOPSY_KIDNEY_TRANSPLANT_REJECTED_VS_OK_DN	1162	132	SELENBP1,MXI1,GATM,CRYAA,CRYAB,TOX3,GRB10,HSD11B2,ABAT,GRB14,ACAA1,ACADSB,AHCY,ERBB4,PTH1R,ALDH4A1,ESRRG,GGH,RAB7L1,ACPP,CYP17A1,UBA1,FGF9,BNIP3,SMPDL3A,SERPINA5,SLC22A6,FGF1,PEPD,GRHPR,CLIC5,MT1E,MT1G,SHMT1,PDZRN3,PCK2,PHYH,MT1X,PLG,PCK1,ACSM3,HPGD,SLC7A	Genes down-regulated in kidney biopsies from patients with acute transplant rejection compared to the biopsies from patients with well functioning kidneys more than 1-year post transplant.	down	0
M3238	SMID_BREAST_CANCER_RELAPSE_IN_BONE_UP	209	28	SLC44A4,ADH1B,CHAD,GATA3,GP2,PLA1A,RAPGEF3,CA12,KIF5C,FBP1,TOX3,CYP2B7P1,ABAT,PSCA,ACADSB,FGFR3,PAH,CYP2B7P1,MUC1,PLK2,HMGCS2,AGR2,MLPH,SERPINA5,SEMA3B,FAM134B,BCAS1,TOM1L1	Genes up-regulated in bone relapse of breast cancer.	down	0
M16932	CHIANG_LIVER_CANCER_SUBCLASS_PROLIFERATION_DN	312	49	ALDH1L1,AQP9,LDHD,SELENBP1,ADH1A,AGXT,AZGP1,AGXT2L1,MAT1A,RGN,DAO,GPT2,ALDH6A1,SERPING1,POR,BHMT,ABAT,SLC38A3,NAGS,OASL,NR1I3,BAAT,MLYCD,CYP8B1,DCXR,CYP2J2,OGDHL,RHOB,HPD,KNG1,GRHPR,ACADL,ADH6,AASS,PIPOX,CLDN14,FMO4,CYP4A22,ADH4,ETNK2,LOC255167,PDK4,PCK1,	Top 200 marker genes down-regulated in the 'proliferation' subclass of hepatocellular carcinoma (HCC); characterized by increased proliferation	down	0

M5408	RODWELL_AGING_KIDNEY_DN	263	30	LDHB,CRABP1,SELENBP1,SLC4A1,GNG7,GABARAPL1,WNK1,TOX3,PALM,EGF,GAD1,RHOBTB3,LPL,GABARAPL1,ESRRG,BMP7,CLDN8,KNG1,NUDT4,PTGER3,PEBP1,OAT,ABTB2,AASS,TMPRSS2,PHYH,BRP44L,MPPED2,PEG3,ZIM2	Genes whose expression decreases with age in normal kidney.	down	0
M5988	REACTOME_SLC_MEDIATED_TRANSMEMBRANE_TRANSPORT	340	47	SLC36A2,SLC6A20,SLC44A4,SLC6A3,SLC9A3,SLC7A8,SLC7A5,SLC16A1,SLC2A5,SLC2A3,SLC2A1,SLC4A1,SLC4A3,SLC34A1,SLC38A1,SLC2A12,SLC38A3,SLC11A1,SLC30A2,SLC6A13,SLC6A19,SLC13A1,SLC13A2,SLC13A3,SLC5A11,SLC5A3,SLC20A2,SLC5A1,SLC26A2,SLC1A4,SLC1A3,SLC43A1,SLC26A6,SLC3	Genes involved in SLC-mediated transmembrane transport	down	0
M3045	CHEMNITZ_RESPONSE_TO_PROSTAGLANDIN_E2_UP	298	35	FBXO17,SARS2,TTK,PTTG1,SLC16A1,EIF4EBP1,ANK2,ASPM,OLFML2A,UHRF1,SPAG5,LMNB1,AFAP1L2,AFAP1L1,TK1,PEA15,STAMBPL1,PVT1,MKI67,DLEU2,TOP2A,NUSAP1,TNFRSF9,KSR1,SLC39A14,GJC1,HJURP,BUB1,SHMT2,FLT1,CENPF,MLF1IP,SMC4,CCNB1,KIF20A	Genes up-regulated in CD4+ [GeneID=920] T lymphocytes after stimulation with prostaglandin E2 [PubChem=5280360] .	up	1.00E-04
M4502	GAL_LEUKEMIC_STEM_CELL_DN	518	86	KIFC1,CADM3,LILRA6,NCF2,SIRPA,C1QA,LILRB1,ZWINT,FER1L4,CX3CR1,LILRB4,PRDM1,VSIG4,CLEC5A,CYP1B1,LMNB1,IFI30,SLC11A1,CD68,TPI1,ALDH4A1,CDC42EP3,LGALS3,LGALS1,LAMA4,ADAP2,DHFR,SERPINA1,CHST15,TOP2A,LYZ,CD163,VCAN,G0S2,GPR65,HMMR,FCGR1B,BUB1,PHLDA1,LILRA6,LIL	Genes down-regulated in leukemic stem cells (LSC)	up	1.00E-04
M4235	GRAHAM_CML_DIVIDING_VS_NORMAL_QUIESCENT_DN	207	29	MPZL2,CRHBP,TSPAN6,TCEAL2,CXCL11,CH25H,IL1B,HLA-DQA1,H1F0,CD40,HLA-DQA1,TFPI,HLA-DPA1,GBP2,TCF4,ADAM28,TNFSF10,CYTIP,EVI2A,HCP5,HLA-DQB1,IFI16,HLA-DQB1,CLEC2B,PTPRC,SELL,BIRC3,ANXA5,SOD2	Genes down-regulated in quiescent CD34+ [GeneID=8842] cells isolated from peripheral blood of normal donors compared to the dividing cells from CML (chronic myeloid leukemia) patients.	up	1.00E-04
M15015	FARMER_BREAST_CANCER_CLUSTER_1	120	24	CYTIP,CD8A,RSAD2,OAS1,IFI44L,OAS2,ISG15,IL10RA,CD2,XAF1,ITK,GIMAP4,CD3D,GZMA,IFI44,IFIT3,GZMK,OASL,CCR5,GMFG,CCR2,LCK,FKBP11,LCP2	Cluster 1: interferon	up	1.00E-04
M4872	TARTE_PLASMA_CELL_VS_PLASMABLAST_UP	897	100	C4A,FSTL1,HOXD10,B2M,RPLP0,CRYAB,GPER,SERPING1,PTPRN,SIRPA,HLA-DQA1,RPS14,HLA-DOB,AVPR1B,PCDHGA10,IL4R,SERPINA3,SERPINA1,HSF4,LYZ,HLA-DQB2,RPL39,PCSK6,PCK1,QPCT,LRP1,DUSP1,CXCL12,TDGF3,CPA3,RELB,FBP1,HLA-F,COL1A1,TNFAIP3,TNFAIP2,SPTB,APOC1,MDK,RPL7A,NR4A1	Genes up-regulated in mature plasma cells compared with plasmablastic B lymphocytes.	up	1.00E-04
M6890	HELLER_HDAC_TARGETS_DN	506	57	ADCY7,CD52,ISG20,RPS19,CCR2,HLA-DRA,HSPA1A,HSPA1B,SERPINH1,TCF4,BLNK,S100A11,CLEC7A,PDIA3,GJA1,MYC,CEACAM1,FMNL1,MYCN,CCND1,CCND2,GPM6B,MYOM2,BCL2,CLEC2B,SHMT2,LRRC41,CD180,QPCT,PBX1,PSAT1,APOBEC3G,PMAIP1,SLC7A5,SLC38A1,FGFR3,LAPTM5,ARHGDIB,ABHD14A,IL21R,	Genes down-regulated in at least one of three multiple myeloma (MM) cell lines by TSA [PubChem=5562] .	up	1.00E-04
M4950	ACEVEDO_LIVER_TUMOR_VS_NORMAL_ADJACENT_TISSUE_UP	1728	96	RPLP0,KIAA1522,SERPINH1,PEA15,RGS5,LYZ,SERPINB1,FOXC1,DEGS1,HLA-C,HLA-B,HLA-F,TCFL5,CCL5,PTTG1IP,UBD,FKBP5,TMED7,COL5A2,APOLD1,BTN3A2,SFXN2,TK1,IDH1,PLA2G4C,CANX,ATP5S,SLC26A6,FKBP11,CXCL9,IL10RB,TOM1L1,CSTB,GBP4,STAMBPL1,KCTD3,SREBF2,FAM84B,CD93,MPV17L,T	Genes up-regulated in liver tumor compared to the normal adjacent tissue.	up	1.00E-04
M2118	SMID_BREAST_CANCER_RELAPSE_IN_PLEURA_DN	83	12	GABBR1,UBD,HLA-DQB1,CXCL9,CEACAM1,HCG26,CHAC1,CCL18,SIX1,CP,FABP7,PSAT1	Genes down-regulated in pleura relapse of breast cancer.	up	1.00E-04
M3015	HUANG_DASATINIB_RESISTANCE_UP	152	25	OSMR,FSTL1,TGFBI,IL15RA,EGFR,KIAA1949,LYN,IFI16,F3,TFPI,BTN3A3,GBP3,BTN3A2,DUSP10,FXYD5,SAMD9L,RAC2,CDC42EP3,ANXA1,PSMB8,PSMB9,IFIT3,ITGA5,PLAU,NNMT	Genes whose expression positively correlated with sensitivity of breast cancer cell lines to dasatinib [PubChem=3062316] .	up	1.00E-04
M17723	CHAUHAN_RESPONSE_TO_METHOXYESTRADIOL_DN	233	35	PDLIM1,MXI1,B2M,TAPBP,RPL12,HADH,TMSB10,HLA-G,TNFAIP3,CCL3,MCL1,ARPC5,CD74,IDH2,ARHGDIB,MKI67,LGALS1,S100A10,UBA1,CYFIP2,MARCKS,SLC1A4,PCP4,ADM,BTG2,PKM2,UBC,TUBB,VIM,ENO2,IL2RG,HCLS1,LSP1,HSP90B1,PGK1	Genes down-regulated by 2-methoxyestradiol (2ME2) [PubChem=1573] in the MM.1S cell line (multiple myeloma) sensitive to dexamethasone [PubChem=5743] .	up	1.00E-04

M17033	LINDSTEDT_DENDRITIC_CELL_MATURATION_A	110	21	TLR2,CXCL11,SLC7A5,CXCL10,ISG15,IL1B,ICAM1,PLA2G7,IL12B,GBP1,FKBP5,NFKBIE,TNC,CCL5,CCL4,CCL20,PLAT,PLEK,STAT1,TNFSF9,SOD2	Maturation of monocyte-derived dendritic cells (DC) in response to inflammatory stimuli: genes up-regulated only at 8 hr after the stimulation (cluster A).	up	1.00E-04
M9343	BOYAULT_LIVER_CANCER_SUBCLASS_G5_DN	56	17	HMGN3,LAPTM5,IL10RA,CLEC2B,TGM2,WIPF1,HLA-DPB1,ARHGDIB,ICAM1,S100A11,IFI44,IFI16,STAT1,SIRPA,RAB31,HLA-DPA1,ARL4C	Down-regulated genes in hepatocellular carcinoma (HCC) subclass G5	up	1.00E-04
M10702	JAEGER_METASTASIS_DN	550	71	PTGS1,DNASE1L3,AQP3,LTB4R,RAB25,TPSB2,S100A2,MATN2,PRELP,KRT19,TACSTD2,ST14,LAMC2,ENPP2,CDA,TPD52L1,ETS2,NTRK2,FCGBP,BMP7,FERMT1,GJA1,TPSAB1,TPSB2,ESRP2,EHF,COL7A1,CD24,MT1M,EPPK1,TRIM29,QPCT,ABCC3,MPZL2,MCOLN3,GATA3,SLC2A1,CPA3,EGFR,LAD1,CA12,CHP2,AKR1B1	Genes down-regulated in metastases from malignant melanoma compared to the primary tumors.	down	1.00E-04
M11837	RODWELL_AGING_KIDNEY_NO_BLOOD_DN	286	31	LDHB,CRABP1,GABARAPL1,FA2H,LRRC2,WNK1,TOX3,FAM171A1,PALM,EGF,GAD1,RHOBTB3,LPL,GABARAPL1,ESRRG,BMP7,CLDN8,BCAN,KNG1,NUDT4,PTGER3,CYB5A,PEBP1,OAT,AASS,TMPRSS2,PHYH,BRP44L,MPPED2,PEG3,ZIM2	Genes whose expression decreases with age in normal kidney	down	1.00E-04
M16473	KEGG_ALDOSTERONE_REGULATED_SODIUM_REABSORPTION	86	11	ATP1B1,FXYD4,ATP1B2,KCNJ1,PIK3R5,SCNN1G,SCNN1B,SCNN1A,ATP1A1,ATP1A2,HSD11B2	Aldosterone-regulated sodium reabsorption	down	1.00E-04
M10501	RODRIGUES_THYROID_CARCINOMA_POORLY_DIFFERENTIATED_UP	1325	70	OSMR,CDCA7,HIST1H2BK,SPIRE1,ZWINT,GNAI1,SERPINH1,HIST1H2BK,LAPTM4B,MKI67,GAS2L3,LEPROTL1,KISS1R,P4HA1,TBC1D7,HIST1H2BE,CCNB1,PSAT1,EIF4EBP1,CDKN2B,SLC2A1,ENC1,NETO2,TNFRSF12A,GINS2,GLT25D1,NBN,NXPH4,NUSAP1,IFI16,GPNMB,ANXA1,TNFSF9,SMC4,IL6ST,GPT2,STC2,LMN	Genes up-regulated in poorly differentiated thyroid carcinoma (PDTC) compared to normal thyroid tissue.	up	2.00E-04
M17788	LINDGREN_BLADDER_CANCER_CLUSTER_1_DN	754	89	KIFC1,EFNA1,PTTG1,CDCA7,CDCA2,PITX2,H1F0,DEPDC1,MGAT1,CGN,ZWINT,PMP22,NEK2,ERBB2,AKAP12,DAG1,TAGLN2,TK1,PEA15,IDH2,LGALS1,CELSR3,MCAM,RAD54L,LAMA4,NTRK2,TCF19,NRP2,FERMT3,PDIA5,PIK3AP1,TOP2A,PEPD,PRG2,SH2D2A,SLC26A6,HSD3B7,BUB1,PCSK6,KIF21B,MT1G,SELL,HCLS	Down-regulated genes whose expression profile is specific to Custer I of urothelial cell carcinoma (UCC) tumors.	up	2.00E-04
M18107	WILENSKY_RESPONSE_TO_DARAPLADIB	56	16	CXCR3,CCL5,HLA-DMA,CD68,EVI2A,HMOX1,EVI2B,CD4,LAIR1,CHI3L1,CTSS,CYBB,ARRB2,CCR2,PLA2G7,BIN2	Atherosclerotic process genes whose coronary expression changed after darapladib [PubChem=9939609] treatment.	up	2.00E-04
M12112	VART_KSHV_INFECTION_ANGIOGENIC_MARKERS_UP	337	58	F2RL3,PTGS2,EFNA1,OSMR,IL6ST,PGF,PTGS1,CXCL11,CXCL12,TGFB1,IL10,MMP1,CXCL10,S1PR1,IL1B,ERAP1,WNT10A,EFNB2,HIF1A,CCR5,F3,DLL4,VEGFA,TNFAIP2,FGFR2,FGFR3,ERBB2,CCL5,TIMP1,ANGPTL1,PLAT,KDR,SFRP2,NOTCH4,GRB7,PLAU,NRP2,NRP1,HEY1,CXCR4,HMOX1,FGF1,ANGPT2,SH2D2A,A	Angiogenic markers up-regulated in lymph endothelial cells upon infection with KSHV (Kaposi's sarcoma herpes virus).	up	2.00E-04
M2200	LEE_DIFFERENTIATING_T_LYMPHOCYTE	299	42	CD52,CD53,AQP3,GATA3,CD3G,CD3D,CECR1,ITGAL,ITGB2,LAPTM5,RAC2,ICOS,UBASH3A,ARHGDIB,ITK,IKZF1,TARP,VAV1,PSMB9,CORO1A,CYFIP2,SASH3,SLA,CXCR4,EVI2A,EVI2B,ARHGEF6,ARHGAP25,IFNAR2,TARP,BTG2,LCK,CD247,TRIM14,RNASET2,CD2,SELPLG,PTPRC,SELL,HCLS1,MAL,GMFG	Genes enriched at every T lymphocyte differentiation stage compared to the early passage fetal thymic stromal cultures (TSC).	up	2.00E-04
M5929	CROONQUIST_STROMAL_STIMULATION_UP	98	17	LUM,CCL4,CTGF,TGFBI,TGM2,THBS2,MYOF,SFTPB,SLC25A4,SPARC,LAMA4,DUSP1,ID1,SULF1,COL1A2,VCAN,NNMT	Genes up-regulated in ANBL-6 cell line (multiple myeloma	up	2.00E-04
M4448	BOYAULT_LIVER_CANCER_SUBCLASS_G23_UP	116	10	SLC26A2,DNAJC6,PKM2,TFPI,SLC38A1,NEK2,MKI67,NDUFA4L2,MLF1IP,ABCG1	Up-regulated genes in hepatocellular carcinoma (HCC) subclass G23	up	2.00E-04
M4844	KEGG_CHEMOKINE_SIGNALING_PATHWAY	382	41	ADCY7,ADCY6,CXCL11,CXCL12,CXCL10,GNG7,CCL4L2,CCR5,ARRB2,CX3CR1,CCR2,CCL3,GNAI1,CCL5,CCL4,RAC2,ITK,VAV1,CXCL16,PREX1,CXCR2,CXCR3,CXCR4,CCL3L1,CCL3L3,SHC1,PLCB2,LYN,WAS,GNB5,GNB4,PRKCZ,CXCL9,GNG11,CCL20,CCL21,PIK3R5,HCK,STAT1,CCL18,JAK3	Chemokine signaling pathway	up	2.00E-04
M10961	GOZGIT_ESR1_TARGETS_DN	1620	173	ISG20,TMEM140,DIRAS2,RAB27B,UGCG,SIPA1L2,TFPI,ABAT,CYP1B1,DUSP10,ARG2,ELF5,SERPINA5,SERPINA3,SERPINA1,PRSS35,CEACAM1,EPB41L1,DEGS2,HLA-DQB1,AOX1,CP,GRIK3,CXCL12,CDKN2B,TGFBI,STMN3,CCL5,CXCL16,ST8SIA4,PTTG1IP,C4A,PMEPA1,EGLN3,PRSS8,XAF1,GPNMB,SCNN1A,COBLL1	Genes down-regulated in TMX2-28 cells (breast cancer) which do not express ESR1 [GeneID=2099]) compared to the parental MCF7 cells which do.	up	3.00E-04

M11840	SHEPARD_BMYB_MORPHOLINO_DN	320	47	GRIK3,TTK,VTN,NR2E1,AQP3,SLC2A5,CDCA2,GPT2,ASPM,EOMES,NR0B2,PTPRN,UHRF1,COL1A2,COL1A1,PMP22,RBP4,PFKFB4,SFXN2,MDK,TK1,FGG,SLC29A2,FGA,LHX1,MKI67,SLC6A13,DHFR,SFRP1,PRODH2,FABP7,KIF23,SYNGR1,HEY1,SARDH,SOX11,GRHPR,SCIN,C3,CEBPA,ATP1A1,PCK1,CCNB1,PYGL,SULF1	Human orthologs of genes down-regulated in zebra fish after knockdown of BMYB [GeneID=4605] by morpholino.	up	3.00E-04
M13763	RADAEVA_RESPONSE_TO_IFNA1_UP	66	16	E2F2,IFITM3,IFI44L,CXCL11,IFI35,CXCL10,VCAM1,BCL2,BST2,KLK3,HLA-C,STAT1,HLA-E,TNFSF10,IFI27,IRF1	Genes up-regulated in primary hepatocytes and Hep3B (hepatocyte) cells in response to IFNA [GeneID=3439] .	up	3.00E-04
M14437	VALK_AML_CLUSTER_5	68	17	SIRPB1,TLR8,EFHD2,VDR,TYMP,NOD2,SMPDL3A,LILRA6,IFNGR2,SIRPA,LILRB1,LILRB2,RASSF4,LILRB3,SIGLEC7,VCAN,SLC15A3	Top 40 genes from cluster 5 of acute myeloid leukemia (AML) expression profile; 96% of the samples are FAB M4 or M5 subtype.	up	3.00E-04
M4308	SATO_SILENCED_BY_METHYLATION_IN_PANCREATIC_CANCER_1	977	131	LDHB,PGF,ISG20,GIT2,TYRO3,GPER,CYP2B7P1,HNF4A,ST14,CYP1B1,DUSP10,KCNJ2,RAC2,TRPV2,ELF5,BNIP3,SERPINA1,ANGPT2,ST6GALNAC2,IGF2,INS-IGF2,KSR1,C3,LRRC31,EPB41L4A,PML,PDK1,QPCT,PSAT1,CXCL12,NR2E1,RNASE2,DNAJC6,CPA3,IL1B,BST2,CHAC1,HLA-C,HLA-B,CDO1,HLA-G,TFAP2C	Genes up-regulated in the pancreatic cancer cell lines (AsPC1	up	3.00E-04
M1262	REACTOME_GRB2_SOS_PROVIDES_LINKAGE_TO_MAPK_SIGNALING_FOR_INTERGRINS_	32	6	APBB1IP,VWF,FGG,FGA,FGB,FN1	Genes involved in GRB2:SOS provides linkage to MAPK signaling for Intergrins	up	3.00E-04
M17693	GARY_CD5_TARGETS_UP	971	78	GNG7,WNT10A,TCF4,SYNGR3,RGS1,P4HA1,KIAA1949,WAS,DOK3,WIPF1,PDK1,SHMT2,LRRC41,ACLY,PCK2,LSP1,PSAT1,DAK,TGFB1,IL10,MFHAS1,SLC2A3,RELB,HLA-E,DDIT4,TNFAIP3,CCNG1,CCL4,CDKN1A,SLC1A4,SLC43A1,LCK,TMC8,TNFSF9,CD52,WWOX,WNK1,LILRB4,VEGFA,ALDOC,CD70,CD74,IDH2,SCD,P	Genes up-regulated in Daudi cells (B lymphocytes) stably expressing CD5 [GeneID=921] off a plasmid vector.	up	4.00E-04
M14877	BASAKI_YBX1_TARGETS_DN	794	78	HLA-DMA,HLA-DMB,IL6ST,FAM110C,PTGS1,MXI1,ISG20,MALL,GBP5,VEGFA,TFPI,STC1,BTN3A3,BTN3A2,GRB14,ALDOC,XIST,CASZ1,LAMB1,SCD,KCNK3,IFI27,GAS2L3,ARSD,STOX1,MARCKSL1,SMPDL3A,P4HA1,SERPINA1,ADM,SERPINB1,RAP1GAP,HK2,RPL37,TPCN1,RNASET2,PCSK6,PHLDA1,PDK1,BTN3A2,BTN	Genes down-regulated in SKOC-3 cells (ovarian cancer) after YB-1 (YBX1) [GeneID=4904] knockdown by RNAi.	up	4.00E-04
M5792	LI_AMPLIFIED_IN_LUNG_CANCER	332	48	RPL17,LDHB,ATP1B1,LDHA,RPL36A,CRABP2,SLC7A5,ENC1,AKR1B10,COL1A2,CSTB,COL1A1,ALDOA,MCL1,ERBB2,MDK,TIMP1,FGG,RPS27,FGB,MSLN,IDH2,LAPTM4B,MUC1,P4HB,RPL23A,PLAU,UCHL1,PRDX4,RPS2,CANX,PFKP,MMP12,UQCRH,PKM2,RPL35,RPL37,MIF,SYN1,HSPA5,THBS2,SCNN1A,ENO1,PLP2,STAT	Genes with increased copy number that correlates with increased expression across six different lung adenocarcinoma cell lines.	up	4.00E-04
M12890	ROZANOV_MMP14_TARGETS_UP	534	68	RARRES3,PTGS1,PDLIM1,ISG20,CDCA7,CTGF,N4BP2L1,SMOX,S100A2,MATN2,NCF2,SIRPA,ALDOC,OAS1,SERPINH1,HLA-DPB1,LAMB1,SLAMF7,APOL3,LAMA4,SRPX2,QPRT,NNMT,SOBP,TNFRSF11B,SERPINA5,CHST15,ERRFI1,MMP14,CCND2,CCL20,HCLS1,SOD3,NUPR1,MAGED4B,TLR2,CSPG4,PMAIP1,KRT86,CA12,	Genes up-regulated in HT1080 cells (fibrosarcoma) over-expressing MMP14 [GeneID=4323] compared to those with knockdown of the gene by RNAi.	up	4.00E-04
M4562	LENAOUR_DENDRITIC_CELL_MATURATION_UP	186	25	ATP1B1,HMGN3,DNASE1L3,SLA,TGFA,GATM,PFKP,CYB5A,MMP12,HLA-DQA1,TNFAIP6,CCND2,PEBP1,HLA-DQB1,RAP1GAP,CD74,TGM2,SCARB1,HLA-DPB1,LPL,IDO1,BIRC3,ABCG1,FCGR2B,DUSP1	Genes up-regulated during in vitro maturation of CD14+ [GeneID=929] monocytes (day 0) into immature (day 7) and mature dendritic cells (day 14).	up	4.00E-04
M12029	BILD_HRAS_ONCOGENIC_SIGNATURE	543	58	HMGN3,PNMA2,PTGS2,FAM110C,MALL,VEGFA,CYP1B1,XIST,LIF,ARG2,AHNAK2,KLF6,GAS1,FERMT1,AGPAT9,TGFA,LYN,MMP14,TNFRSF10B,SERPINB1,G0S2,HK2,FAM46B,CYP27B1,CCL20,MEG3,PHLDA1,FLRT3,TBX3,DUSP1,TOB1,SH2D5,SLC2A3,DNAJC6,IL1B,ELOVL7,PTHLH,LRAT,FGFR2,LOC400931,MCL1,TNFR	Genes selected in supervised analyses to discriminate cells expressing activated HRAS [GeneID=3265] oncogene from control cells expressing GFP.	up	4.00E-04
M2540	KYNG_DNA_DAMAGE_BY_GAMMA_AND_UV_RADIATION	82	12	NRP2,CRHBP,VCAM1,FMO4,PLCL1,SLC1A3,CXCR4,EVI2A,IL10RB,LOX,CCND1,NNMT	Gamma and UV responding genes.	up	4.00E-04
M3218	MOSERLE_IFNA_RESPONSE	62	17	IFIH1,RTP4,RSAD2,IFI44L,OAS1,OAS2,SAMD9L,CMPK2,CXCL10,IFI44,IFI16,STAT1,IFIT3,IFIT2,OASL,TNFSF10,GBP1	Top 50 genes up-regulated in ovarian cancer progenitor cells (also known as side population	up	4.00E-04

M15425	VANTVEER_BREAST_CANCER_ESR1_DN	448	57	LDHB,MALL,KCNK5,LILRB3,ST14,VGLL1,CTSL2,NFKBIE,CHI3L1,S100A10,RAD54L,LYN,ADM,FOXC1,NMI,NMB,BUB1,TRIM29,CD180,CCL18,SOD2,PHGDH,PSAT1,DUSP9,TTK,SLC2A5,IL15RA,SAMD4A,FNDC3B,PDXK,LAD1,TMSB10,HIF1A,CSTB,TNFAIP3,FAM171A1,GBP1,ASS1,ST8SIA1,ITGB2,CCL5,NDRG1,DEFB1	Down-regulated genes from the optimal set of 550 markers discriminating breast cancer samples by ESR1 [GeneID=2099] expression: ER(+) vs ER(-) tumors.	up	4.00E-04
M4491	CROONQUIST_NRAS_VS_STROMAL_STIMULATION_DN	166	20	GJA1,CTGF,TGFBI,ARHGAP11A,SLC25A4,COL1A2,VCAN,TPM1,TK1,LAMB1,THBS2,SFTPB,RAD51AP1,MKI67,SPARC,CCNB1,LAMA4,DUSP1,ID1,SULF1	Genes down-regulated in ANBL-6 cell line (multiple myeloma	up	4.00E-04
M16441	REACTOME_INTEGRIN_CELL_SURFACE_INTERACTIONS	164	23	VTN,RAPGEF3,ICAM1,LAMC3,COL1A2,COL1A1,ITGAL,TNC,ITGB2,APBB1IP,VCAM1,FGG,FGA,FGB,LAMB1,FN1,COL4A3,ITGA2,ITGA4,VWF,ITGA5,PECAM1,ITGAD	Genes involved in Integrin cell surface interactions	up	4.00E-04
M15472	VECCHI_GASTRIC_CANCER_EARLY_DN	811	97	LDHB,AQP4,AGXT2L1,SOSTDC1,FAM150B,GNG7,GPER,KIF5C,LIFR,CD36,CCR2,ABAT,CHGB,SLC2A12,CYP1B1,GCNT2,ENPP2,PCDH20,HLA-DOB,ESRRG,SLAMF7,KLF15,GAS1,SFRP1,MAP9,KCNJ16,KCNJ15,FAM3B,SOBP,BNIP3,ATP4B,GIF,CHRDL1,GRIP2,C7,LOC400043,FHL1,MFSD4,GPM6B,FAM46C,MT1E,WIPF3,M	Down-regulated genes distinguishing between early gastric cancer (EGC) and normal tissue samples.	down	4.00E-04
M244	SMID_BREAST_CANCER_RELAPSE_IN_BRAIN_DN	183	20	IL6ST,MLPH,SLC44A4,SLC7A8,SELENBP1,GSTM3,SERPINA5,GATA3,C4A,RAB27B,CA12,TOX3,ST6GALNAC2,SLC27A2,ERBB4,DACH1,BIK,TJP3,MAP9,AGR2	Genes down-regulated in brain relapse of breast cancer.	down	4.00E-04
M9206	YAMASHITA_LIVER_CANCER_STEM_CELL_DN	152	20	ALDH1L1,MASP1,CDKN2A,F11,KNG1,ALDH6A1,APCS,PPARGC1A,HNF4A,SLC27A2,FKBP5,FMO4,HRG,ABCB1,NR1I3,ABCG5,AOX1,CYP8B1,CD14,UGT2B7	Genes down-regulated in hepatocellular carcinoma (HCC) cells with hepatic stem cell properties.	down	4.00E-04
M17778	DIAZ_CHRONIC_MEYLOGENOUS_LEUKEMIA_DN	250	45	CRHBP,TLR2,CD53,ISG20,GATA3,TGFBI,BCR,CECR1,HLA-C,HLA-B,CST7,CCR2,LILRB4,MNDA,CSTA,CLEC5A,MVP,MCL1,IFI30,OAS2,BLNK,LPL,RPL23A,ADAP2,NKG7,HLA-DMB,SLC1A3,RPL41,BTG2,FCGR1A,FCGR1C,SGCB,RPL39,IL10RA,MYOF,MS4A6A,GAPDH,MS4A4A,PTPRE,RPL18A,SELL,ANXA5,LILRA6,LILR	Genes down-regulated in CD34+ [GeneID=947] cells isolated from bone marrow of CML (chronic myelogenous leukemia) patients	up	5.00E-04
M7284	SEITZ_NEOPLASTIC_TRANSFORMATION_BY_8P_DELETION_UP	159	29	RARRES3,TLR4,IFI44L,CXCL11,CXCL10,ISG20,MALL,ISG15,CH25H,EVI2B,EFNB2,IFI44,COL1A2,GBP1,IFIH1,CLU,OAS1,OAS2,DAAM1,CCL5,GPRC5A,BCHE,CD24,ADAM28,IDO1,IFIT3,IFIT2,IFI27,OASL	Genes up-regulated in CT60/4 cells (breast cancer reverted to normal by transfer of chromosome 8p region) vs parental MDA-MB-231 cells (deleted chromosome 8p).	up	5.00E-04
M4120	LOPEZ_MBD_TARGETS	1748	132	LDHA,RPLP0,PDHA1,SERPING1,SERPINH1,SLC29A2,SERPINA5,SERPINA1,ERRFI1,PARVG,LYZ,C7,SORBS1,LRRC41,AXL,CDH16,HLA-B,CSDA,HLA-E,HLA-F,IFITM3,NR4A1,CDKN1C,CDKN1A,PTTG1IP,FABP1,SDC3,PAX8,APLN,SLC43A1,SLC43A3,CA9,PKM2,RAGE,EHD2,SELPLG,CEBPA,SECTM1,MAL,CD53,C1QC,AL	Genes up-regulated in HeLa cells (cervical cancer) after simultaneus knockdown of all three MBD (methyl-CpG binding domain) proteins MeCP2	up	5.00E-04
M999	HADDAD_T_LYMPHOCYTE_AND_NK_PROGENITOR_DN	139	23	SCN3A,SLC2A5,LYZ,TARP,CST7,ZWINT,VCAN,G0S2,CSTA,NPTX2,CD24,MS4A6A,BLNK,TYROBP,PLEK,HCK,LGALS1,RNASE6,TARP,RAB31,CCL3,CCL3L1,IRF8	Genes down-regulated in hematopoietic progenitor cells (HPC) of T lymphocyte and NK (natural killer) lineage.	up	5.00E-04
M3261	KEGG_TOLL_LIKE_RECEPTOR_SIGNALING_PATHWAY	206	20	TLR1,TLR2,TLR3,TLR4,CXCL11,TLR7,TLR8,CXCL10,IL1B,CD40,CCL3,CCL5,CCL4,LBP,IFNAR2,IL12B,CXCL9,PIK3R5,STAT1,CD14	Toll-like receptor signaling pathway	up	5.00E-04
M15662	WATANABE_ULCERATIVE_COLITIS_WITH_CANCER_DN	30	8	GBP5,PREX1,SLA,NLRC5,BTN3A1,GBP4,GBP1,LCP2	Genes down-regulated in non-neoplastic rectal mucosa samples from patients having cancer associated with ulcerative collitis	up	6.00E-04
M7928	MITSIADES_RESPONSE_TO_APLIDIN_UP	987	67	HIST1H2AD,ISG20,HIST1H2BK,LRRC2,PEA15,PPP1R3C,ARG2,HLA-DRB6,HLA-DOA,RECQL5,MARCKS,BHLHE41,KSR1,PHLDA1,KCNE4,PHYH,FEM1C,DUSP1,AZGP1,HLA-E,COL1A1,NFE2L2,C3AR1,IFITM3,TIMP1,VDR,TNFSF10,PIP5K1B,RHOB,HCP5,LCK,TSC22D3,SCNN1A,FLT1,TNFSF9,PPARGC1A,CCR2,CHKA,OAS1,	Genes up-regulated in the MM1S cells (multiple myeloma) after treatment with aplidin [PubChem=44152164]	up	6.00E-04
M15940	NAKAMURA_METASTASIS_MODEL_DN	88	16	IRX3,C3,ALDOC,SFXN3,EHBP1L1,AQP3,CALCA,RAC2,PLAT,UGCG,EOMES,PTHLH,TFPI,DDB2,SERPINB1,PMP22	Top genes up-regulated in subcutaneous tumors from highly metastatic pancreatic cancer cells.	up	6.00E-04

M3444	REACTOME_TRANSMEMBRANE_TRANSPORT_OF_SMALL_MOLECULES	438	51	SLC36A2,SLC9A3,SLC2A12,SLC11A1,SLC29A2,SLC28A1,SLC20A2,SLC26A2,G6PC,SLC26A6,SLC39A14,HK2,SLC4A9,SLC15A4,SLC15A3,SLC6A20,SLC44A4,SLC6A3,SLC7A8,SLC7A5,SLC16A1,SLC2A5,SLC2A3,SLC2A1,SLC4A1,SLC4A3,SLC34A1,SLC38A1,SLC38A3,SLC30A2,SLC6A13,SLC6A19,SLC13A1,SLC13A2	Genes involved in Transmembrane transport of small molecules	down	6.00E-04
M9936	PAPASPYRIDONOS_UNSTABLE_ATEROSCLEROTIC_PLAQUE_UP	111	19	MPEG1,CSF2RB,FCGR3A,FCGR3B,CTSS,HLA-G,CD163,CD84,MLEC,PLA2G7,RSAD2,IFI30,RNASET2,IDH1,MS4A4A,NCKAP1L,CCL18,SOD2,IFIT3	Genes up-regulated in unstable ateroslerotic plaques compared to the stable ones.	up	7.00E-04
M9673	ZHOU_INFLAMMATORY_RESPONSE_LPS_UP	908	84	PTGS2,LTBP4,ISG15,CH25H,SCN2B,SSTR2,MAPK4,RTP4,HSPA1A,AHNAK2,SLC28A1,MARCKS,G0S2,IL12B,ARL4D,CCL20,MT1G,MXRA5,CCL18,RNF19B,ADH1B,CXCL11,GLYATL1,CXCL10,MOBKL2A,IL1B,ELOVL7,RELB,HSPB7,TNFAIP2,TM4SF18,CCL4,ANGPTL1,NBN,SLFN5,CCRL2,FRMD7,EFCAB4B,TNFSF9,IFIT3,I	Genes up-regulated in macrophages by P.gingivalis LPS (lipopolysaccharide).	up	7.00E-04
M9143	FRIDMAN_SENESCENCE_UP	156	32	HTATIP2,MMP1,CDKN2A,CDKN2B,ISG15,CTGF,RHOB,CRYAB,IFI16,CCND1,F3,IGSF3,COL1A2,MDM2,TFAP2A,TNFAIP3,TNFAIP2,CYP1B1,VIM,ESM1,PEA15,HSPA2,FN1,S100A11,SPARC,STAT1,CDKN1C,RAB31,CDKN1A,SMPD1,IGFBP2,IGFBP3	Genes up-regulated in senescent cells.	up	7.00E-04
M3652	DER_IFN_ALPHA_RESPONSE_UP	116	20	PMAIP1,ISG15,PDXK,BST2,HLA-A,HLA-C,IFI16,HLA-E,GBP1,NMI,TRIM14,PML,OAS1,OAS2,IFI35,TAP1,STAT1,TRIM21,IFIT3,IRF1	Genes up-regulated in HT1080 cells (fibrosarcoma) by treatment with interferon alpha for 6 h.	up	7.00E-04
M19745	TARTE_PLASMA_CELL_VS_PLASMABLAST_DN	631	52	LDHB,KIFC1,PDLIM1,CD52,GLDC,GIT2,LILRB4,AHCY,LMNB1,NEK2,IFI30,CD70,IDH2,MKI67,LGALS1,GGH,S100A10,TOP2A,GZMB,DHFR,CLIC1,CCND2,HMMR,CLEC2B,SELL,CD180,SOD2,LSP1,CCNB1,YWHAH,PGK1,HPGD,TTK,SLC7A5,SLC16A1,TMSB10,MVP,ARHGDIB,ARID3A,PSMB8,CDKN1A,CORO1A,KIF23,CYTI	Genes down-regulated in mature plasma cells compared with plasmablastic B lymphocytes.	up	7.00E-04
M13984	MARZEC_IL2_SIGNALING_UP	216	29	TNFSF14,CXCL12,IL10,TGFB1,AHR,CCL3,TNFRSF12A,HSPA1B,WT1,LIF,PEA15,PTPN7,TNFSF10,ACVR1B,CSF2RB,RHOB,MYC,TNFRSF9,SERPINB9,CCND1,CCND2,HK2,TRIB3,TNFRSF1B,BCL2,IL10RA,PHLDA1,IL2RB,BIRC3	Genes up-regulated by IL2 [GeneID=3558] in cells derived from CD4+ [GeneID=920] cutaneous T-cell lymphoma (CTCL).	up	7.00E-04
M12480	LINDSTEDT_DENDRITIC_CELL_MATURATION_B	96	17	NBN,CYTIP,MARCKSL1,CXCR4,TAP1,IL15RA,TNIP1,CSF2RA,LYN,RELB,BIRC3,SLAMF1,TNFRSF9,ADM,MARCKS,TNFAIP3,TNFAIP2	Maturation of monocyte-derived dendritic cells (DC) in response to inflammatory stimuli: genes up-regulated both at 8 hr and 48 hr after the stimulation (cluster B).	up	7.00E-04
M2761	NAKAYAMA_SOFT_TISSUE_TUMORS_PCA2_UP	183	24	NRP2,CRABP1,FGF9,SLC7A5,TOP2A,ASPM,PITX1,NUSAP1,FGFR3,NEK2,TNC,HMMR,COL9A3,HJURP,NPTX2,BUB1,ENO2,AHNAK2,PLP2,HS3ST3A1,MKI67,CENPF,CCNB1,KIF20A	Top 100 probe sets contrubuting to the positive side of the 2nd principal component; associated with adipocytic differentiation.	up	7.00E-04
M16036	CHANDRAN_METASTASIS_UP	226	20	SLC7A5,SLC16A1,TYRO3,WNK1,TMSB10,DDIT4,VEGFA,NGF,HSPA1A,PVT1,P4HA1,LYN,VCAN,HSPA1A,HSPA1B,GRAMD4,HSPA6,HS6ST1,THBS2,NUPR1	Genes up-regulated in metastatic tumors from the whole panel of patients with prostate cancer.	up	7.00E-04
M12101	REACTOME_P130CAS_LINKAGE_TO_MAPK_SIGNALING_FOR_INTEGRINS	32	6	APBB1IP,VWF,FGG,FGA,FGB,FN1	Genes involved in p130Cas linkage to MAPK signaling for integrins	up	7.00E-04
M9911	WOO_LIVER_CANCER_RECURRENCE_DN	162	31	ACOX2,PPARA,CYP2J2,ALDH1L1,ADH1B,MAT1A,DAO,PCYT2,SARDH,HPD,SUOX,CYB5A,SERPING1,POR,G6PC,HNF4A,ACAA1,ACADSB,ADH6,AASS,ECHS1,PAH,PIPOX,FMO4,ACY1,PCK2,MTTP,CYP4F3,CYP4F2,DCXR,PC	Genes negatively correlated with recurrence free survival in patients with hepatitis B-related (HBV) hepatocellular carcinoma (HCC).	down	7.00E-04
M12596	SETLUR_PROSTATE_CANCER_TMPRSS2_ERG_FUSION_DN	42	5	AQP2,KLHL21,MPPED2,ITGAD,CSDC2	Genes down-regulated in prostate cancer samples bearing the fusion of TMPRSS2 with ERG [GeneID=7113;2078].	down	7.00E-04
M8451	SENESE_HDAC3_TARGETS_UP	1040	79	SERPINE2,SPIRE1,GRB10,PMP22,ESM1,ERRFI1,FMNL2,MMP14,CCND2,FOXC1,ARL4C,CCL20,TBC1D4,PHLDA1,HAPLN1,PDK4,CXCL10,SLC2A3,STMN2,ENC1,TCFL5,AKR1B10,TNFAIP3,TM4SF18,IL32,ZNF697,CDKN1C,ACOX2,E2F7,SLFN5,RHOB,IFI16,ANXA4,IFIT3,IFIT2,SLC14A1,EFNA1,IL6ST,PRDM1,SAA2,KL	Genes up-regulated in U2OS cells (osteosarcoma) upon knockdown of HDAC3 [GeneID=8841] by RNAi.	up	8.00E-04

M11585	NAGASHIMA_NRG1_SIGNALING_UP	367	35	EDN1,PMAIP1,AQP3,ISG20,GATA2,CTGF,AKIRIN1,VEGFA,LAMC2,MCL1,TNFRSF12A,DUSP10,LIF,ARG2,FBXO41,CDC42EP3,KLF6,NR4A1,HOMER1,ETS2,FERMT2,TNFRSF11B,CXCR4,AEN,SEMA3B,MYC,TNFRSF10B,ADM,CAMK2G,EGLN3,PHLDA1,FLRT3,EPPK1,DUSP1,ID1	Genes up-regulated in MCF7 cells (breast cancer) after stimulation with NRG1 [GeneID=3084] .	up	8.00E-04
M2777	AMIT_SERUM_RESPONSE_240_MCF10A	122	14	OCLN,TLR2,TGFA,GBP1,ABLIM3,AKAP12,PRSS8,PGBD5,CD24,PHLDA3,ANGPTL4,ITGA2,CDKN1C,ITGA5	Genes whose expression peaked at 240 min after stimulation of MCF10A cells with serum.	up	8.00E-04
M1918	WEST_ADRENOCORTICAL_TUMOR_UP	609	46	PTTG1,ZWINT,STC1,NEK2,SLC31A2,SCD,GGH,HOMER1,MCAM,ANGPT2,TOP2A,IGF2,INS-IGF2,EPB41L4B,BUB1,PCSK6,ATP1A1,ACLY,CCNB1,ACSM3,KIF20A,CEP76,PMAIP1,SLC7A5,SLC16A1,TBC1D13,DNAJC6,ASPM,FGFR1OP,TNFAIP3,IL32,PSMB7,HOXA9,RHOBTB3,SLC1A3,TUBA1B,TUBA1C,STOM,NPTX2,GAPDH,	Up-regulated genes in pediatric adrenocortical tumors (ACT) compared to the normal tissue.	up	8.00E-04
M19261	YAGI_AML_WITH_T_8_21_TRANSLOCATION	824	72	ADCY7,BTK,APOB,SERPINE2,PRAME,BCR,HLA-DQA1,TFPI,TPSAB1,TPSB2,HLA-DPA1,HLA-DRA,PALM,ACADSB,CD74,HLA-DPB1,MKI67,LGALS1,UBA1,SASH3,LEPROTL1,ARHGEF6,CCND1,CDR1,TPSAB1,TPSB2,PTGDS,HLA-DQB1,GPM6B,ACSM3,NUPR1,FBLN5,HLA-DRB1,AGXT,CDKN2C,MS4A2,CSF3R,CIITA,SOCS3,CS	Genes specifically expressed in samples from patients with pediatric AML (acute myeloid leukemia) bearing t(8;21) translocation.	up	8.00E-04
M13266	KEGG_RENAL_CELL_CARCINOMA	142	10	PGF,ARNT2,TGFB1,SLC2A1,TGFA,HIF1A,VEGFA,EGLN3,PIK3R5,ETS1	Renal cell carcinoma	up	8.00E-04
M625	REACTOME_CHEMOKINE_RECEPTORS_BIND_CHEMOKINES	112	20	CXCR2,CXCR3,CXCL11,CXCL12,CXCL10,CCL3L1,CXCR4,CXCR7,CCL3L3,CCRL1,CCR5,CX3CR1,CCR2,CCL3,CXCL9,CCL5,CCL4,CCL20,CCL21,CXCL16	Genes involved in Chemokine receptors bind chemokines	up	8.00E-04
M6744	COLDREN_GEFITINIB_RESISTANCE_DN	461	55	RAB25,KIAA1522,KRT19,PITPNM3,CGN,TACSTD2,ST14,ATPIF1,LAMC2,EPS8L1,SLC2A12,GCNT2,PVRL4,BLNK,CNKSR1,LGALS9,ACPP,SRPX2,AGR2,CYP2J2,NPNT,RAB11FIP4,ANKRD22,DOK7,ESRP2,EHF,DAPP1,EPPK1,TMPRSS4,BIK,TJP3,PRSS22,USP44,OCLN,MPZL2,LAD1,ERP27,GRHL1,OVOL2,PPL,ARHGDIB,A	Genes down-regulated in NSCLC (non-small cell lung carcinoma) cell lines resistant to gefitinib [PubChem=123631] compared to the sensitive ones.	down	8.00E-04
M13183	ODONNELL_TARGETS_OF_MYC_AND_TFRC_UP	175	26	RARRES3,MPEG1,PTGS1,SLC2A3,IL4R,HLA-DQB1,BTNL9,LYZ,HLA-DQA1,CX3CR1,ABAT,LCP2,FUT6,FAM46C,LLGL2,TSC22D3,HLA-DOB,ZBP1,KLF6,MYO1G,KLK1,ANXA4,LGALS9,RGS1,ALDH2,C1RL	Genes down-regulated in P493-6 cells (B lymphocyte	up	9.00E-04
M15664	ROSTY_CERVICAL_CANCER_PROLIFERATION_CLUSTER	282	34	KIFC1,TTK,PTTG1,MCM10,EIF4EBP1,CDKN2A,ASPM,TMSB10,SPAG5,ZWINT,SLC38A1,NETO2,LMNB1,NEK2,PAQR4,TK1,MKI67,GGH,CELSR3,DHFR,KIF23,E2F8,TOP2A,NUSAP1,HMMR,HJURP,BUB1,RAD51AP1,CENPF,KIF18B,MLF1IP,SMC4,CCNB1,KIF20A	The 'Cervical Cancer Proliferation Cluster' (CCPC): genes whose expression in cervical carcinoma positively correlates with that of the HPV E6 and E7 oncogenes; they are also differentially expressed according to disease outcome.	up	9.00E-04
M11720	BEIER_GLIOMA_STEM_CELL_DN	146	16	HLA-DMA,HLA-DMB,HLA-DRB1,HLA-DMB,HLA-DQB1,HLA-DQA1,CCND1,FAM167A,HLA-DPA1,HLA-DRA,BTN2A2,CD74,LAPTM5,GPNMB,MORN4,IGFBP3	Genes down-regulated in cancer stem cells derived from glyoblastoma tumors: CD133+ [GeneID=8842] vs. CD133- cells.	up	9.00E-04
M15476	URS_ADIPOCYTE_DIFFERENTIATION_UP	124	19	MASP1,APOB,LBP,ALDH6A1,CRYAB,MAP4K3,COL1A2,HSD11B2,TNFAIP2,FXYD1,AGT,TAP1,PLCD1,GPD1,LPL,PLEK,SCD,IGFBP2,FABP7	Genes up-regulated in primary adipocytes compared to preadipocytes.	up	9.00E-04
M7272	KEGG_PARKINSONS_DISEASE	268	8	SLC6A3,NDUFA4L2,UBE2L6,GPR37,UBA1,UCHL1,SLC25A4,UQCRH	Parkinson's disease	up	9.00E-04
M12289	REACTOME_PEPTIDE_LIGAND_BINDING_RECEPTORS	348	47	F2RL3,EDN1,CXCL11,CXCL12,CXCL10,GPER,SSTR2,CCR5,F2,CX3CR1,CCR2,C3AR1,CCL3,BDKRB1,BDKRB2,CCL5,CCL4,NPY5R,NPY,CXCL16,AVPR1B,AVPR1A,SST,CXCR2,CXCR3,EDNRA,KISS1R,CXCR4,CCL3L1,CXCR7,CCL3L3,APLN,CCRL1,KNG1,AVPR2,C3,CXCL9,FPR3,NMB,APLNR,CCL20,CCL21,AGT,ANXA1,NPY	Genes involved in Peptide ligand-binding receptors	up	9.00E-04
M9450	REACTOME_PLATELET_ADHESION_TO_EXPOSED_COLLAGEN	30	8	LYN,ITGA2,VWF,GP6,CD36,COL1A2,FCER1G,COL1A1	Genes involved in Platelet Adhesion to exposed collagen	up	9.00E-04
M205	WEST_ADRENOCORTICAL_TUMOR_MARKERS_UP	49	11	NEK2,IL32,PMAIP1,NPTX2,STC1,IGF2,INS-IGF2,PCSK6,TOP2A,ASPM,KIF20A	Top up-regulated genes in pediatric adrenocortical tumors (ACT) compared to the normal tissue.	up	0.001

M18058	WILCOX_PRESPONSE_TO_ROGESTERONE_UP	327	33	HMGN3,CEP76,CDKN2C,ASPM,PTHLH,TNFAIP6,TK1,PPL,IDH1,KLF6,SCD,ABCG1,NPY,NKG7,KCNJ16,SYNPO2L,EVI2A,SMPDL3A,HMOX1,TOP2A,CCRL1,NUSAP1,IFNAR2,HMMR,HJURP,PCSK6,SHMT1,MT1M,CENPF,NPY1R,MLF1IP,CCNB1,KIF20A	Genes up-regulated in primary cultures of ovarian surface epithlium cells exposed to progesterone [PubChem=5994] for 5 days.	up	0.001
M17730	GRAHAM_CML_QUIESCENT_VS_NORMAL_DIVIDING_UP	145	22	PTGS2,PMAIP1,CXCL11,CXCL10,HIST1H2BK,IL1B,TPSB2,DDIT4,TPSAB1,TPSB2,GBP1,CYP1B1,XIST,APOC1,CCL5,CCL20,HIST1H2BK,SOD2,RGS1,CCL3,CCL3L1,TPSAB1	Genes up-regulated in quescent CD34+ [GeneID=8842] cells isolated from peripheral blood of CML (chronic myeloblastic leukemia) patients compared to the dividing cells from normal donors.	up	0.001
M6444	NUYTTEN_NIPP1_TARGETS_UP	1382	101	PTGS2,TMEM140,ISG20,ACTG2,ISG15,HIST1H2BK,CTGF,GBP5,GRB10,RTP4,MST1,TCF4,APOL2,PARP9,CYP2J2,BNIP3,P4HA1,FGF1,MMP13,G0S2,HIST1H2AD,PML,HK2,ITM2C,CCL20,DGKD,MT1E,TBC1D7,WIPF1,MT1G,FLRT3,PDK1,MT1X,ACCN2,DUSP1,SH2D5,SAMD4A,VAMP4,TNFAIP3,NETO2,NR4A1,TNFSF10,NB	Genes up-regulated in PC3 cells (prostate cancer) after knockdown of NIPP1 [GeneID=5511] by RNAi.	up	0.001
M3026	CHUNG_BLISTER_CYTOTOXICITY_UP	294	29	CNDP2,C1QC,ALDH6A1,CD3D,GNLY,SERPING1,HCST,C1QB,CCR5,TNFAIP3,TPI1,HAVCR2,TIGIT,KLRC1,KLRC2,CDKN1A,ADAP2,MIR155HG,CD8B,SLC1A4,SLC43A3,GZMB,CYB5A,DDB2,PLIN2,MT1E,MT1F,PCK2,PHYH	Genes up-regulated in blister cells from patients with adverse drug reactions (ADR).	up	0.001
M19630	ZHU_CMV_ALL_UP	122	16	NAMPT,SLC7A5,CXCL10,ISG15,HLA-E,GBP1,SLC39A14,PML,OAS1,OAS2,CCL5,ENO2,NR4A1,IDO1,TRIM21,IFI27	Up-regulated at any timepoint following infection of primary human foreskin fibroblasts with CMV	up	0.001
M19667	CHENG_RESPONSE_TO_NICKEL_ACETATE	58	6	SLA,MS4A6A,MAP1B,APOL1,ABCC3,CP	Genes down-regulated in HPL1D cells (lung epithelium) upon exposure to nickel acetate [PubChem=9756] .	up	0.001
M18334	REACTOME_CLASS_A1_RHODOPSIN_LIKE_RECEPTORS	586	66	EDN1,S1PR1,LTB4R,S1PR5,HTR1F,GPER,SSTR2,CCR5,LPAR6,CCR2,HTR6,F2,CX3CR1,BDKRB1,BDKRB2,NPY5R,NPY,AVPR1B,AVPR1A,EDNRA,KISS1R,CCL3L1,CCL3L3,AVPR2,C3,CXCL9,GPR65,FPR3,NMB,CCL20,CCL21,AGT,NPY1R,PTGFR,P2RY13,F2RL3,CXCL11,CXCL12,CXCL10,C3AR1,CCL3,CCL5,CCL4,P2RY8,	Genes involved in Class A/1 (Rhodopsin-like receptors)	up	0.001
M17002	REACTOME_BILE_SALT_AND_ORGANIC_ANION_SLC_TRANSPORTERS	26	5	SLC16A1,SLC13A1,SLC13A2,SLC13A3,SLC47A2	Genes involved in Bile salt and organic anion SLC transporters	down	0.001

[a]Systematic name assigned by Moleclar Signatures Database

[b]The number of differential expressed genes between ccRCC normal and tumor samples

[c]Genes differential expressed between Tumor and normal samples with adjust p-value < 0.05 are regarded as contributing gene

[d]Brief description of gene set

[e]up:up regulation of gene set; down: down-regulation of gene set

[f]adjustment is based on Benjamini-Hochberg method

Supplementary Figure 1. RNAseq reveals known gene expression, pathway, and chromosome changes in ccRCC Heatmap of top 10 up/down regulated plus selected genes in ccRCC tumor and normal tissues. The scales of each color are showed in the top.

Supplementary Figure 2. Heatmap of gene expression level in RUNX1-RUNXT1 gene set. (A) Heatmap of 66 differential expressed RUNX1-RUNXT1 related genes, revealed by eight paired ccRCC tumor and normal RNA-seq data. 54 genes up-regulated and 12 genes down-regulated in ccRCC samples. (B) Validation of differential expressed with microarray data consist of 90 ccRCC and 13 normal kidney tissues, similar patten was found campared to result from RNA-seq data. Genes involed in the heatmap from top to bottom are: NETO2, GBP2, VCAN, SLC1A3, PLK2, SERPINA1, TRIB3, IFI16, SLC2A5, LILRB1, GPNMB, CD163, TLR7, OAS2, KLF6, TLR2, NDRG1, TMEM176A, COL1A2, C3AR1, MYOF, STAT1, SIRPA, LAIR1, SLC15A3, NUPR1, TMEM176B, MALL, ENPP2, PHLDA1, VAT1, VSIG4, IFI44, CD180, SOCS3, FNDC3B, CXorf21, CLEC5A, UPP1, ANXA2, SCG5, DNASE2, JAG2, SLAMF8, LAMC1, CD1D, JUN, PTP4A3, SPATS2L, TUBB6, OLR1, BTG1, EMP1, DHRS9, CRHBP, SLC5A3, PALM, LPL, PLAT, ID1, UCHL1, QPRT, ACVR1B, CH25H, HOXA9, TMEM110.

Supplementary **Figure 1.**

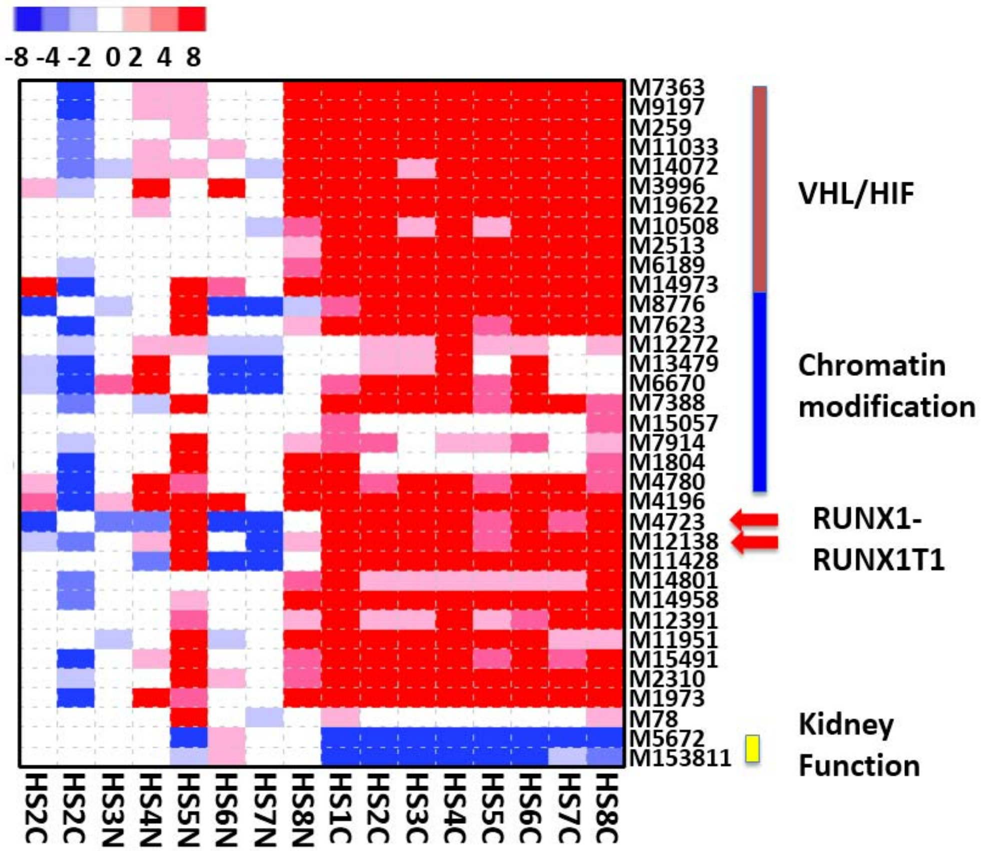

Supplementary Figure 2.

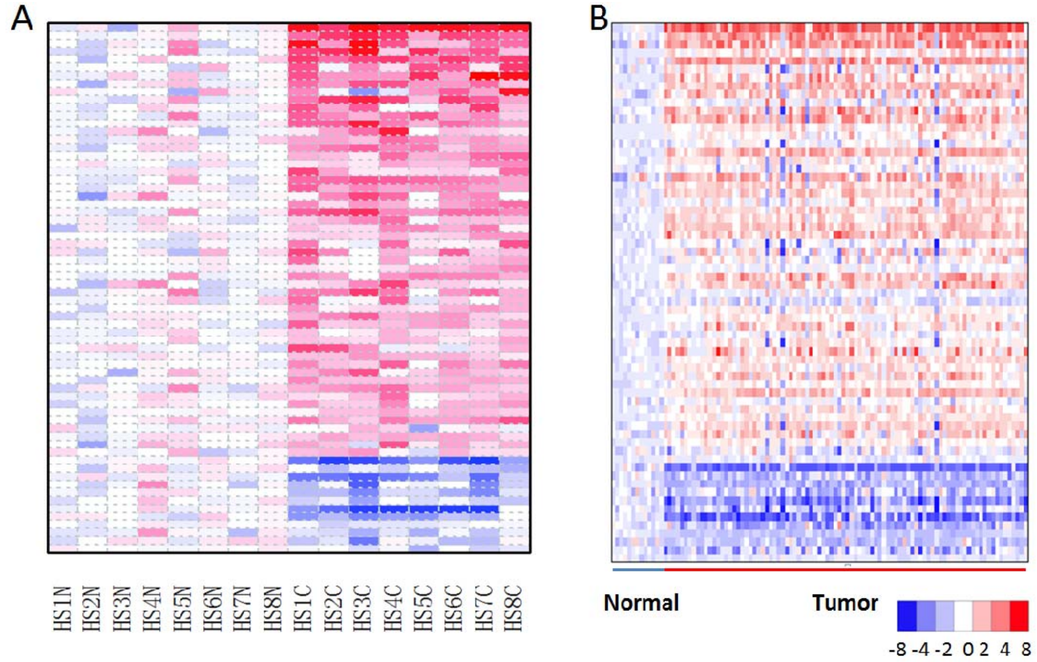

Supplement: Supplementary file 1 — All the patients listed in the table were treated with radical nephrectomy (RN) for RCC at Huashan Hospital of Fudan University. The tissues experimented in RNA-seq test were from the first 8 cases. And we used the tissues for all 16 cases in qRT-PCR examination. [file 450621.f1.pdf]
